# Pressure-sensitive composite bandages with high toughness, self-healability and strong tissues adhesion for rapid bone fixation and accelerated osteogenesis via immunomodulation and neovascularization

**DOI:** 10.1016/j.mtbio.2025.102730

**Published:** 2025-12-23

**Authors:** Puzhou Lei, Shuya Wang, Kaiwen Chen, Linghanqing Wang, Yi Shen, Huize Zhong, Sida Chen, Xin Su, Yu Zhao, Huanan Wang, Lei Li

**Affiliations:** a Department of Orthopedics, Shengjing Hospital of China Medical University, Shenyang, 110004, PR China; b Key State Laboratory of Fine Chemicals, School of Bioengineering, Dalian University of Technology, Dalian, 116024, PR China; c Department of Orthopedic Surgery, Peking Union Medical College Hospital, Chinese Academy of Medical Sciences and Peking Union Medical College, Beijing, 100032, PR China

**Keywords:** bone adhesive, Citric acid-based polymer, Hydroxyapatite, Pressure-sensitive adhesives, Osteogenic composite

## Abstract

Bone adhesives have gained considerable interest as an adjunctive approach for fragment fixation in comminuted fractures and bone graft procedures. However, the current adhesives are mostly used to fill the bone void to achieve the fixation of broken bones, which inevitably impedes regeneration at the fracture interface. To address this limitation, we present an integrated bone-adhesive bandage that enables robust adhesion to bone tissue without occluding the healing space. Precise modulation of the viscoelastic properties in a poly(octamethylene citrate)–gelatin (POC-G) copolymer facilitates strong interfacial adhesion to bone but also unique internal cohesion to enable self-heal. Further incorporation of hydroxyapatite (HA) nanoparticles significantly enhances the adhesive strength (570.43 ± 21.82 kPa) while preserving mechanical toughness (1648.66 ± 367.81 kJ m^−3^). This strategy further renders POC-G/HA composites with the unique ability to achieve instant, repeatable, and sustained bone fixation in a humid environment, addressing a critical limitation of conventional commercial adhesives. Beyond serving as a superior mechanical fixative, this multifunctional bandage actively orchestrates the bone regeneration process. It promotes the osteogenic differentiation of stem cells, modulates macrophage polarization towards a pro-regenerative M2 phenotype, and stimulates neovascularization to enhance local nutrient and cell supply. This breakthrough design of a pressure-sensitive bone-adhesive bandage opens a new avenue for the instant structural integration of bone fragments and the fixation of bone grafts during surgery. It represents a paradigm shift from passive fixation to active regeneration, potentially revolutionizing therapeutic strategies for bone repair.

## Highlights

• An integrated bone adhesive bandage with high pressure-sentitive adhesion, mechanical toughness and biological activity.
• Immediate, repeatable, and durable fixation in moist environments without compromising the bone healing area.
• Stem cell osteogenic differentiation, macrophage polarization toward the M2 phenotype, and angiogenesis.
• Clinical applications in fracture fixation and bone reconstruction.

## Graphical abstract

**Figure:**
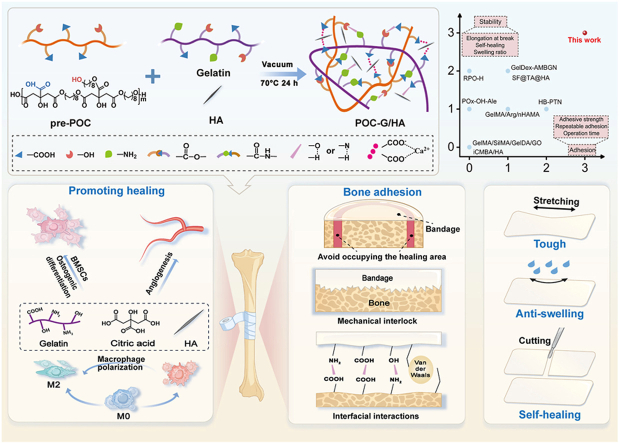

## 1 Introduction

Bone fractures represent one of the leading global traumatic injuries, imposing a substantial socioeconomic burden through direct healthcare costs and indirect productivity losses [1,2]. Current clinical interventions primarily rely on metallic implants (e.g., titanium plates, intramedullary nails) for rigid fixation to restore initial mechanical stability [3]. Subsequent osseointegration between newly formed bone and the implant enables gradual transfer of load-bearing capacity from the device to the reconstructed bone [4,5]. Crucially, intimate contact between bone tissues and fragments (or grafts) at the injury site is essential to facilitate regeneration and prevent compromised healing outcomes, such as delayed union or nonunion [[6], [7], [8]]. Nevertheless, achieving stable bone-to-bone apposition in complex, highly comminuted fractures remain a formidable clinical challenge. To address this, bone adhesives have recently emerged as a promising solution, most of which employ different polymerization schemes to realize in situ solidification and fixation, physically locking the fractured bones [9,10]. Typical examples include cyanoacrylate (CA), polyurethane (PU), and polymethyl methacrylate (PMMA)-based adhesives, which have been clinically applied for bone adhesion [11,12]. However, these commercial adhesives face significant challenges in orthopedic applications, such as the cytotoxicity during in situ polymerization, compromised adhesion strength in the presence of blood or body fluids [13,14], and critically, non-degradability or cytotoxic degradation products that induce prolonged local inflammation and consequently impair osteogenic efficacy [15]. Thus, developing novel bone adhesives that achieve robust interfacial adhesion under wet physiological conditions for reliable fragment fixation during initial application, while maintaining mechanically stable load transfer throughout bone regeneration via osteogenic activity, remains a formidable challenge.

Hydrogels capable of bone adhesion can supplement or even replace traditional bone adhesives due to their similarity to the extracellular matrix (ECM), biocompatibility, low immune response, and biodegradability—making them suitable for bone integration. To develop functional bone adhesives, hydrogel-based systems typically employ adhesive group functionalization (e.g., catechol moieties, aldehyde groups, succinimide esters) for robust interfacial bonding under physiological conditions, combined with multi-network composite designs (e.g., dual-network or biphasic hard/soft structures) to enhance mechanical strength. For instance, Cui et al. developed a bone adhesive hydrogel by oxidizing dextran to generate aldehyde groups that form Schiff base linkages with bone collagen via reactions with gelatin and aminated bioactive glass (BG) [16]. The inorganic BG component augmented mechanical strength and osteogenic activity, enabling stable bone adhesion and healing of comminuted fractures. However, this hydrogel's compressive modulus (∼80 kPa) remains substantially below the requirement for clinical bone adhesives. To address mechanical robustness limitations, Liu et al. engineered a biphasic hydrogel bone adhesive comprising a rigid phase of phosphorylated polyglutamic acid (P-PGA) encapsulating tetracalcium phosphate (TTCP), covalently integrated with a viscoelastic soft phase of amino-functionalized PEGylated poly(glycerol sebacate) and P-PGA [17]. This system achieved an adhesive strength of ≈280 kPa and compressive modulus of ≈1.02 MPa, meeting load-bearing orthopedic fixation requirements for comminuted fractures. Nevertheless, the enhanced crosslinking density and strength that improve matrix properties simultaneously slow in vivo degradation rates, hindering synchronization with new bone ingrowth. Consequently, developing bone adhesives that simultaneously deliver high adhesion strength, osteogenic activity, and minimal physical obstruction to new bone formation remains critical for clinical translation.

**Scheme 1:**
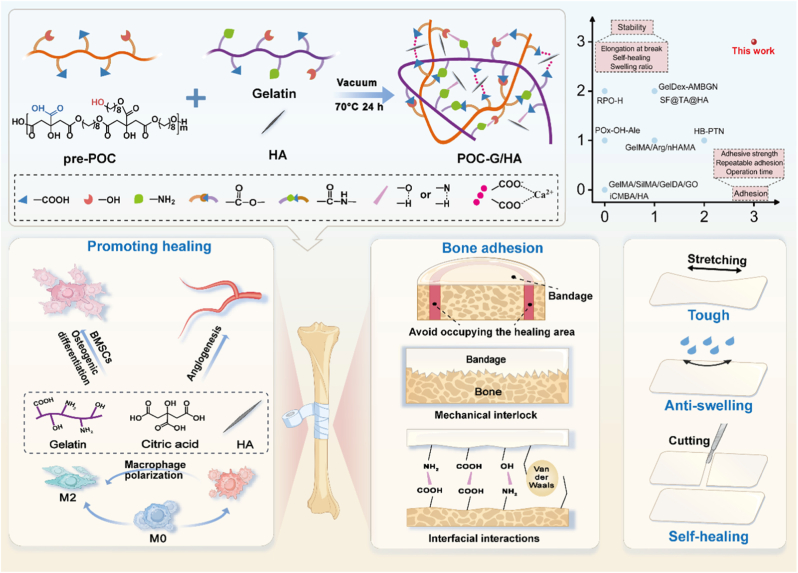
Schematic illustration of the fabrication and application of the composite bone adhesive bandage, highlighting its role in promoting bone regeneration through osteogenic differentiation of stem cells, modulation of the immune microenvironment, and stimulation of angiogenesis.

Adhesive bandages provide fixation of bone defects through surface adhesion while avoiding physical occupancy of the healing region, thereby eliminating obstruction to new bone formation. However, conventional hydrogel-based adhesive patches face limitations in bone adhesion due to insufficient availability of tissue-reactive groups and inadequate bulk mechanical strength. Recently, polymer melt-derived pressure-sensitive adhesives (PSAs) have demonstrated exceptional performance by combining dual advantages. Firstly, the high viscoelasticity of the polymer melt can facilitate bulk deformation under light pressure, ensuring local adaptability and intimate substrate contact for instantaneous wetting, while their inherent mechanical robustness can allow efficient energy dissipation to enhance adhesion. Secondly, high-density physical interactions (hydrogen bonding, van der Waals forces, mechanical interlocking) can enable adhesion independent of surface chemistry, allowing attachment to both hydrophilic and hydrophobic substrates [18]. Critically, the reversible nature of these bonds enables detachment with minimal residual interference without compromising the regenerative space. However, traditional polymer melt-based PSAs have been largely confined to skin or non-biological applications due to their inherent hydrophobicity and compromised biocompatibility. Recent state-of-the-art work has addressed this by incorporating hydrophilic PEG molecules into hydrophobic polyester polymers, achieving effective wet tissue adhesion through hydration effects—a strategy that introduces hydrophilic materials for robust adhesion to aqueous tissue interfaces [19]. Despite these advances of previously reported bio-adhesives, an ideal biomaterial for bone adhesion remains elusive, which was expected to simultaneously fulfill the following requirements including high adhesion strength, reversible detachment capability, low swelling ratio, superior biocompatibility, osteogenic activity, and non-interference with new bone formation (Scheme 1, Table S1).

In this study, we developed a novel class of pressure-sensitive bone adhesive bandages composed of a highly adhesive poly (octamethylene citrate) (POC)/gelatin co-polymer network and hydroxyapatite (HA) nanoparticles as reinforcement fillers but also osteogenic components (denoted as POC-G/HA) (Scheme 1). Specifically, we prepared amphiphilic copolymers by interpenetrating biodegradable POC prepolymers (pre-POC) with hydrophilic gelatin macromolecules through thermal polymerization, followed by incorporating osteogenic HA nanoparticles to obtain a functional organic/inorganic composite bone adhesive. The POC-G/HA adhesives can form strong, repositionable adhesions to bone tissues under physiological wet conditions through abundant carboxyl group-initiated hydrogen bonds and intrinsic pressure-sensitive viscoelasticity, meanwhile maintaining structural stability with a substantially low swelling ratio. These outstanding mechanical and handling properties can be attributed to the strong cohesive POC-gelatin interactions and physical HA-polymer matrix interactions. Its multiple reversible interactions simultaneously endow the adhesive bandage with self-healing properties. We further demonstrated the composite bone adhesives possessed strong adhesion to fractured bone tissues in a rat circular cranial bone defect model, but also were capable to promote regeneration of the defective bones by inducing osteogenic differentiation of stem cells, modulating macrophage polarization toward a pro-regenerative M2 phenotype, and stimulating the formation of vasculature to enhance local nutrient and cell supply. In general, our newly designed pressure-sensitive bone adhesive bandage has opened up a new approach for the immediate structural integration of bone fragments and fixation of bone grafts during surgeries, and has the potential to bring about significant changes to the treatment strategies for bone repair.

## 2 Experimental methods

### 2.1 Materials

All chemicals were received in their as-received state and were not subjected to any purification processes. Anhydrous citric acid, 1, 8-octanediol, gelatin, and hydroxyapatite are purchased from Aladdin Co., Ltd. Ex vivo animal tissues were procured from local markets.

### 2.2 Synthesis of bone adhesive bandage

First, poly(octandiol citrate) (POC) prepolymer was modified as reported in previous studies [20]. Anhydrous citric acid (19.21 g, 1 eq) and 1,8-octadiol (14.62, 1 eq) were introduced into a three-necked flask. The mixture was melted by stirring in an oil bath at 160 °C. Subsequently, the system was cooled to 130 °C and stirred for 2 h under a nitrogen atmosphere to facilitate the formation of prepolymers. To eliminate unreacted monomers and oligomers, the resulting product was dissolved in an excess of distilled water. Following centrifugation, the supernatant was discarded, and the residue was dried to remove residual water, yielding yellow transparent viscous liquid.

To prepare the POC-G and POC-G/HA adhesive bandages, the POC prepolymer was dissolved in 70 % ethanol solution to obtain a final mass fraction of 60 wt%, Subsequently, gelatin was added (POC/gelatin, 100/5) for the preparation of POC-G. For the POC-G/HA composites, varying amounts of hydroxyapatite (HA) were incorporated into the mixture. The resulting solution was heated to 60 °C, followed by thorough stirring to ensure homogeneity and complete evaporation of water, yielding a viscous solution. This solution was then cast into a Teflon mold (dimensions of 50 mm × 50 mm × 1 mm) and reaction under vacuum at 70 °C for 24 h to obtain the adhesive bandages. Finally, the adhesive bandages containing 20 wt% and 40 wt% hydroxyapatite (HA) were designated as POC-G/HA 20 and POC-G/HA 40, respectively.

The SEM image shows that hydroxyapatite is needle-like (Fig. S12A). TEM statistical analysis confirmed particle lengths ranging from 104 to 298 nm, with an average length of 190.29 ± 44.34 nm (Fig. S12B and C). In addition, the DLS measurement indicates that the hydrodynamic size of HA is 202.47 nm, and PDI is 0.304 (Fig. S12D). XRD analysis further verified the well-crystallized structure of HA, with diffraction peaks closely matching the standard hydroxyapatite pattern (JCPDS No. 09–0432) (Fig. S12E).

### 2.3 Synthetic characterization

Proton nuclear magnetic resonance spectroscopy (^1^H NMR, Bruker Ascend 600 MHz) was employed to characterize the chemical structure of POC prepolymers dissolved in dimethyl sulfoxide-d6 (DMSO-*d*_6_). Adhesive bandages were analyzed using Fourier transform infrared spectroscopy (FTIR, Thermo) within the wavenumber range of 500–4000 cm^−1^. Additionally, gel permeation chromatography (GPC, Agilent PL-GPC220) was utilized to determine the molecular weight and polydispersity index of the POC (eluent: tetrahydrofuran, standard: polystyrene). X-ray diffraction experiments were carried out using a diffractometer (XRD, MiniFlex 600) equipped with Cu Kα radiation. The 2θ data were collected over the angular range of 5°–80° with a step size of 0.01°. Thermal analysis of the adhesive bandages was conducted using differential scanning calorimetry (DSC, DSC300). Each sample, weighing 15 mg, was analyzed over a temperature range of −60 °C–150 °C at a heating rate of 10 °C/min.

### 2.4 Rheological study of bone adhesive bandage

The bone adhesive bandages were prepared and subsequently punched into circular discs with a diameter of 20 mm. The rheological characteristics were examined using a rotational rheometer (DHR-2). Specifically, the adhesive patch was positioned under a testing jig (20 mm in diameter and a 1 mm gap) at a controlled temperature of 25 °C. Frequency sweeps were conducted within the range of 0.1–100 rad/s under a constant strain of 0.5 %.

### 2.5 Mechanical properties of bone adhesive bandage

The adhesive bandages were subjected to tensile testing using an electronic mechanical testing machine equipped with 50 N force sensors. Dumbbell-shaped adhesive specimens (n = 4) were prepared for the tensile tests, which were conducted at speed of 25 mm/min. The tensile strength and elongation at break were recorded. The tensile strength was defined as the peak stress value recorded prior to material fracture.

### 2.6 In vitro swelling and degradation tests of bone adhesive bandage

The adhesive bandages were punched into a disc with a diameter of 5 mm using a punch and subsequently weighed to record the initial mass (m_1_). The disc was then immersed in an excess of phosphate buffer solution, incubated in an oven at 37 °C for a predetermined time period, and subsequently removed. Excess solution on the surface was carefully blotted with filter paper, after which the disc was reweighed to obtain the final mass (m_2_). The swelling rate of the bonded patch was determined using Equation (1). Four parallel measurements were conducted for each sample set to ensure data reliability. Additionally, images were captured to visually assess the degree of material swelling.

(1) $$\text{Swelling}\;\text{ratio}\;\left(\%\right)=\left(m_{2}‐m_{1}\right)/<m_{1}\times 100\%$$

The adhesive patch, with a diameter of 5 mm, was carefully weighed to determine its initial mass (x_1_). It was subsequently immersed in 30 mL of PBS and the solution was refreshed every 2 days. After being incubated in an oven at 37 °C for a predetermined time period, the patch was retrieved, rinsed thoroughly with distilled water, dried to a constant weight, and reweighed to obtain the final mass (x_2_). The pH of the immersion solution was recorded, and the mass loss rate was then calculated using equation (2). Four parallel measurements were conducted for each sample set to ensure data reliability.

(2) $$\text{Mass}\;\text{lose}\;\left(\%\right)=\left(x_{1}‐x_{2}\right)/x_{1}\times 100\%$$

### 2.7 Adhesive properties of the bone adhesive bandage

Before the adhesive test, the bovine bones were processed as follows. First, the soft tissue on the surface was removed to avoid interference with interfacial adhesion. The bones were then cut into uniform pieces 25 mm long and 10 mm wide.

The adhesive properties of the adhesive bandages were evaluated through lap shear tests using bovine bone slice. Adhesive bandages were affixed to the ends of bone slice, the adhesion region was also defined as a rectangular area measuring 10 mm in length and 10 mm in width. We simultaneously tested the adhesive strength of the adhesive on dry and wet bone surfaces. The dried bone slices were taken out of the PBS solution, with surface moisture blotted using filter paper, and the wet bovine bone slices did not undergo further surface treatment. The crosshead speed was 25 mm min^−1^ after pressing for 20 s at 10 N was used otherwise mentioned. Four parallel tests were conducted for each set of samples to ensure reliability. The adhesive strength of the adhesive patch was evaluated on various tissues and in diverse solutions using this method. The shear strength was calculated according to the following equation:

(3) $$\text{Adhesive}\;\text{strength}=F/W_{1}\times L_{1}$$

Where *F* is the maximum force during shearing, and W_1_ and L_1_ are the width and length of the adhesion area respectively.

### 2.8 Biomineralization of adhesive bandage

The adhesive patch (100 mg) was accurately weighed and subsequently immersed in 50 mL of simulated body fluid (SBF-1.5 × ). It was then placed in a constant temperature water bath maintained at 37 °C, with the SBF being refreshed daily. On the 3rd and 7th days, the adhesive bandages were retrieved, cleaned thoroughly with distilled water to remove any surface contaminants, and dried under controlled conditions to eliminate residual moisture. The extent of mineralization was characterized using scanning electron microscopy (SEM), Fourier-transform infrared spectroscopy (FTIR), and inductively coupled plasma mass spectrometry (ICP-MS).

### 2.9 Cell biocompatibility testing

Bone marrow mesenchymal stem cells (rBMSCs) were extracted from the femoral bone marrow of 3-week-old Sprague-Dawley (SD) rats and maintained in T25 cell culture flasks (Thermo Fisher Scientific, USA). The cells were cultured in conditioned medium (Dulbecco's Modified Eagle Medium (DMEM) supplemented with 10 % fetal bovine serum and 1 % penicillin-streptomycin (PS)) by combining different materials' extracts, under standard conditions of 37 °C and 5 % CO_2_ in a humidified environment. Third-passage rBMSCs were utilized for subsequent experiments. To evaluate biocompatibility, rBMSCs were plated at a concentration of 2 × 10^4^ cells/mL in 96-well plates and incubated with liquid extracts of various groups. The CCK-8 assay (Solarbio, China) was employed to measure cell viability on days 1, 3, and 5, providing insights into the cytotoxic effects of the materials. Additionally, a Calcein/PI cell viability assay kit (Beyotime, China) was utilized for further cytotoxicity analysis. For hemolysis assessment, suspensions of red blood cells were exposed to each material group at 37 °C for 4 h. Following centrifugation, the absorbance of the supernatant was recorded at 577 nm.

### 2.10 In vitro osteogenic experiments

The rBMSCs were plated in 12-well plates and allowed to adhere overnight. Subsequently, the growth medium was substituted with osteogenic induction conditioned medium. Alkaline phosphatase (ALP) activity was analyzed by treating the cells with a BCIP/NBT ALP detection kit (Solarbio, China). Following a 21-day differentiation period, calcium deposition was evaluated through alizarin red (AR) staining. Both ALP and AR staining outcomes were examined via light microscopy. For osteogenic gene expression analysis, RT-PCR was performed. Total RNA was isolated from rBMSCs after 14 days of differentiation and reverse-transcribed into cDNA. Transcript levels of *ALP*, *BMP2*, and *Runx-2* were measured using SYBR Premix Ex *Taq*II (primer sequences in Table S2), with GAPDH (Proteintech, USA) serving as the reference gene. In parallel, Western blotting was conducted to assess protein expression. Cellular proteins were extracted, quantified, and separated by SDS-PAGE electrophoresis. After being transferred to PVDF membranes, samples were blocked with QuickBlock Buffer (Beyotime, China) for 30 min. Membranes were probed with primary antibodies at 4 °C overnight, followed by incubation with HRP-conjugated secondary antibodies at room temperature for 30 min. Protein bands were detected using an Amersham ImageQuant 800 imaging system (Cytiva, USA), and band intensities were quantified with ImageJ software for statistical analysis.

### 2.11 In vitro angiogenesis assay

The Matrigel matrix (Beyotime, China) was uniformly coated onto 96-well plates and polymerized at 37 °C for 1 h. Human umbilical vein endothelial cells (HUVECs, 1 × 10^4^ cells/mL) were co-cultured subsequently with angiogenic induction conditioned medium. Tube formation was imaged and quantified for branch points and tube length.

### 2.12 In vitro macrophage differentiation assay

RAW264.7 cells were plated in 24-well plates and allowed to adhere for 4 h under standard culture conditions (37 °C). The culture medium was replaced with fresh medium containing 100 ng/mL lipopolysaccharide (LPS). The cells were subsequently subjected to immunofluorescence staining using iNOS and Arg-1 antibodies (Proteintech, USA), followed by imaging with a confocal microscope and quantitative analysis of positive cell percentages.

### 2.13 ELISA detection of cell culture supernatant

Microplate wells were initially coated with a specific antigen (Thermo Fisher Scientific, USA) and incubated at 4 °C overnight. Following three washes, unbound sites were blocked with BSA blocking buffer and incubated at 37 °C for 1–2 h, after which another washing step was performed. Test samples and standards were then added and incubated at 37 °C for 1–2 h, followed by additional washing. An enzyme-labeled secondary antibody was subsequently added and incubated at 37 °C for 1 h. After thorough washing, a chromogenic substrate was added and the reaction was allowed to proceed at room temperature in the dark for 10–30 min. Upon color development, the reaction was terminated by addition of a stop solution. Finally, absorbance (OD value) was measured using a microplate reader, and the concentration of target molecules in samples was calculated based on the standard curve.

### 2.14 In vivo degradation and biocompatibility

Male Sprague-Dawley (SD) rats (6–8 weeks old, 200–220 g) underwent isoflurane anesthesia, followed by subcutaneous implantation of experimental materials after disinfection. Explants were harvested and weighed at 2-, 4-, and 8-week post-surgery to assess in vivo degradation rates based on pre- and post-implantation mass differences. Peri-implant skin tissues were processed for hematoxylin & eosin (H&E) and immunofluorescence analyses. At the 4-week timepoint, six rats per group were euthanized for systemic biocompatibility evaluation, with major organs (heart, liver, spleen, lungs, kidneys) collected for H&E staining. The degradation rate was calculated using the formula: Residual weight (%) = [(W_0_ - W_1_)/W_0_] × 100 %. W_0_ and W_1_ represent initial dry weight and post-explantation weight, respectively.

### 2.15 SD rat ring-shaped cranial defect model

Male Sprague-Dawley rats (6–8 weeks, 200–220 g) underwent isoflurane anesthesia for cranial defect modeling. The surgical site was prepared via hair removal and antiseptic treatment. After creating an annular defect in the skull of each rat using a trephine with an outer diameter of 5.5 mm and an inner diameter of 4 mm, the internal bone fragment was carefully removed and subsequently repositioned to its original site. In the CA adhesive group, POC-G, POC-G/HA 20, and POC-G/HA 40 bone adhesive groups, the internal bone fragments were bonded to the surrounding bone tissue using the respective materials, while defects in the control group remained untreated without any material application. Rats were euthanized at 1, 2, 4, and 8 weeks postoperatively for quantitative analysis. Cranial specimens underwent micro-CT scanning (SkyScan1276, Bruker, USA; 70 kV/200 μA) with bone mineral density thresholds set to 89–255 Hounsfield units (HU). A standardized annular region of interest (ROI: 5.5 mm outer/4 mm inner diameter) was reconstructed using CTan software for bone morphometric quantification. Key parameters including bone volume fraction (BV/TV) and trabecular number (Tb.N) were calculated with the defined ROI.

### 2.16 Cranial tissue staining

Following decalcification, the cranial specimens were subjected to histological staining to evaluate bone regeneration and matrix organization. Cranial specimens were fixed in 4 % paraformaldehyde (72 h), decalcified in 14 % EDTA (pH 7.4, 4 weeks), and paraffin-embedded. Coronal sections (2.5 μm) underwent sequential histological staining: H&E and Masson's trichrome staining for neocortical bone morphology. Runx-2 immunohistochemistry to monitor osteogenic regeneration. Multiplex immunofluorescence co-staining was performed with Proteintech (USA) antibodies to simultaneously evaluate macrophage polarization dynamics and angiogenic progression. Macrophage subpopulations were differentiated using phenotypic markers: M1 pro-inflammatory subset (CD68+/iNOS+) versus M2 pro-regenerative subset (CD68+/CD163+). Concurrently, vascular networks were mapped through dual detection of endothelial markers CD31 (pan-endothelial) and EMCN (specific to type H vessels), enabling functional vessel identification critical for osteogenic coupling.

### 2.17 Statistical analysis

The results of the experiments were expressed as the mean and standard deviation (Mean ± SD). At least three experiments were conducted for each experiment. Origin 2021 software and IBM SPSS version v19.0.0 (IBM, America) were used to conduct unpaired t-tests and one-way analysis of variance (ANOVA) with Bonferroni's test. A p-value <0.05 was statistically significant. Statistical significance thresholds were defined as: ∗p < 0.05, ∗∗p < 0.01, ∗∗∗p < 0.001.

## 3 Results and discussion

### 3.1 Design and synthesis of the bone adhesive bandage

**Fig. 1:**
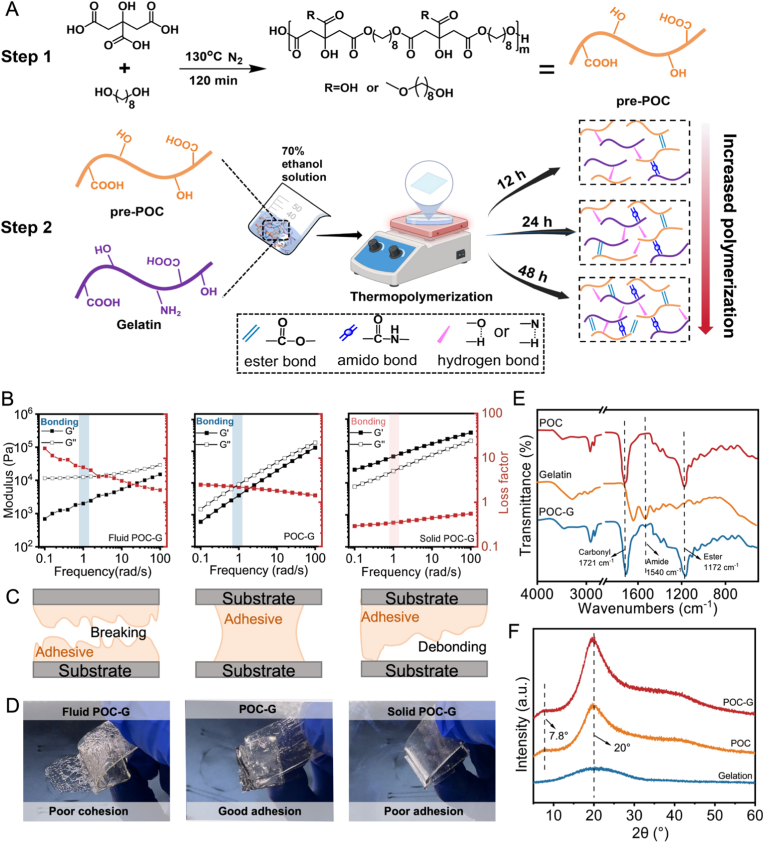
Synthesis and characterization of POC-G. (A) Schematic of the preparation process and structure of the POC-G. (B) Viscoelastic and (C) adhesion properties of POC-G under different reaction times (left, viscous fluid, reaction time was 12 h; middle, viscoelastic POC -G, reaction time was 24 h; right, solid, reaction time was 48h) via frequency sweep. At 1 rad/s, fluid POC-G and POC-G can adhere to the substrate, which is indicated by the blue shading, while solid POC-G cannot adhere to the substrate and is indicated by the red shading. (D) Photographs of the POC-G with different moduli exhibiting viscous fluid (left, reaction time ≈ 12 h), viscoelastic POC-G (middle, reaction time ≈ 24 h), and solid (right, reaction time ≈ 48h) like behaviors. (E) FTIR spectra; and (F) X-ray diffractograms of POC, gelatin, and POC-G.

Our bone adhesive bandage is composed of an entangled polymer network functionalized with adhesive moieties and reinforced by hydroxyapatite (HA) ceramic nanoparticles to achieve strong bone adhesion and osteogenic activity (Fig. 1A). Specifically, poly(1,8-octanediol-co-citrate) (POC) was synthesized from 1,8-octanediol and citric acid, serving as a hydrophobic backbone with a tunable entanglement network. The abundant carboxyl groups on the POC side chains enable extensive interactions with surrounding tissues, thereby enhancing adhesion. Both POC and HA nanoparticles are well recognized for their excellent osteogenic potential and appropriate biodegradability, making them attractive for biomedical applications [[21], [22], [23]]. Moreover, the abundant pendant carboxyl groups of POC facilitated the formation of high-density reversible interactions, including hydrogen bonding and van der Waals forces, with diverse substrates such as bone tissue. To further improve hydration capacity and promote cellular adhesion, hydrophilic, naturally derived gelatin was incorporated, providing bioactive motifs such as RGD sequences that enhance cell attachment [24].

To prepare the composite polymer of POC and gelatin (denoted as POC/G), it is important to note that gelatin cannot directly participate in melt condensation copolymerization with citric acid and 1,8-octanediol, because its molecular chains undergo irreversible denaturation at high temperatures. Therefore, an amphiphilic copolymer network was constructed through a two-step strategy, in which pre-polymerized POC (pre-POC) was subsequently combined with gelatin to form the POC/G copolymer through thermal polycondensation reaction (Fig. 1A). Pre-POC was first synthesized via a polyesterification reaction between 1,8-octanediol and citric acid monomers at 130 °C under a nitrogen atmosphere, with the reaction time carefully controlled between 1 and 3 h to obtain pre-POC of different molecular weights [25]. Shorter reaction times (<1 h) produced low-molecular-weight products with insufficient viscosity, whereas extended polymerization (>3 h) resulted in excessive crosslinking and insolubility. An intermediate polymerization time of 2 h was therefore selected, yielding pre-POC with a tunable molecular weight suitable for subsequent processing. The resulting products were characterized by ^1^H NMR, which confirmed the successful synthesis of pre-POC (Fig. S1A) [26]. Gel permeation chromatography revealed a weight-average molecular weight (Mw) of 1324 g mol^−1^ and a polydispersity index (PDI) of 2.1 (Fig. S1B), consistent with previous reports demonstrating the reproducible synthesis of pre-POC [16]. To ensure homogeneous blending with gelatin and HA nanoparticles, an ethanol–water mixed solvent was employed for dissolving pre-POC. This solvent system not only improved the solubility and processability of pre-POC but also enhanced its compatibility with both the gelatin and inorganic HA nanoparticles, thereby facilitating uniform dispersion during composite fabrication.

To fabricate POC/G copolymers with tunable viscoelastic properties suitable for pressure-sensitive adhesion, pre-POC was firstly blended with gelatin in an ethanol-water solution which ensured the complete dissolution of the polymer (Fig. S2). followed by secondary thermally induced polymerization at 70 °C to form a bandage in the mold. The reaction time of this secondary polymerization step was systematically varied (12, 24, and 48 h) to modulate the degree of polymerization of the resulting networks. The chemical characteristics of the POC/G copolymers were then examined. Differential scanning calorimetry (DSC) revealed a gradual increase in the glass transition temperature (*T*_*g*_) with extended polymerization times, shifting from −23.6 °C to −5.9 °C (Fig. S3). This upward shift in *T*_*g*_ reflected a progressive reduction in chain mobility as the degree of polymerization increased. Fourier transform infrared (FTIR) spectroscopy further confirmed the successful formation of POC-G. Distinct absorption bands were observed at 3500 cm^−1^ (–OH stretching), 2850 and 2930 cm^−1^ (asymmetric and symmetric CH_2_ stretching), 1720 cm^−1^ (ester C=O stretching), and 1170 cm^−1^ (C–O stretching). In addition, the C=O stretching vibration red-shifted from 1721 cm^−1^ in POC to 1710 cm^−1^ in POC-G, suggesting the establishment of strong hydrogen bonding between POC and gelatin. Amide I and II bands appeared at 1650 cm^−1^ and 1470 cm^−1^, confirming the successful incorporation of gelatin into the copolymer network (Fig. 1E). X-ray diffraction (XRD) analysis further substantiated the enhanced intermolecular interactions between POC and gelatin. Pure gelatin exhibited a characteristic diffraction peak at 2θ = 20° [27], while pure POC showed two broad peaks at 2θ = 20° and 7.8°. After copolymerization, the diffraction peak of POC-G at 2θ = 20° became sharper and more intense, indicating strengthened chain packing and intermolecular associations within the composite network (Fig. 1F) [28].

To regulate the PSA properties of the composite bandages, the polymerization time of pre-POC and gelatin mixtures was varied to control the molecular weight of the resulting network. Since the direct determination of molecular weight is impractical after crosslinking, rheological measurements were employed to evaluate the viscoelastic behavior of the polymers synthesized under different polymerization times, thereby realizing the characterization of polymerization degree in an indirect way. The viscoelasticity of polymers is a critical factor in governing PSA performance, which is dictated by the loss factor (tan δ, the ratio of loss modulus G″ to storage modulus G′). Specifically, G″ facilitates surface wetting and interfacial adhesion, while G′ resists shear forces and contributes to cohesion [29]. By systematically adjusting the polymerization time, we optimized the viscoelasticity of the POC-G amphiphilic copolymer to achieve pressure-sensitive adhesion. As the polymerization time increased, the loss factor gradually decreased, and the macroscopic behavior transitioned from a viscous fluid to a viscoelastic solid (Fig. 1D), as further confirmed by rheological analysis (Fig. 1B–S4). At 12 h of polymerization at 70 °C, POC-G displayed a viscous fluid-like behavior (G' < G″, tan δ = 6.43 ± 0.64 at 1 rad/s), which enabled interfacial adhesion but lacked structural stability. In contrast, after 48 h of polymerization, POC-G exhibited predominantly elastic behavior (G' > G″, tan δ = 0.54 ± 0.25 at 1 rad/s), thereby losing its pressure-sensitive property. Importantly, the intermediate sample polymerized for 24 h at 70 °C exhibited balanced viscoelasticity (G' < G″, tan δ = 1.97 ± 0.28 at 1 rad/s), achieving an optimal compromise between cohesion (G' = 4.39 ± 0.21 kPa) and adhesion (G'' > G′ during bonding). Moreover, POC-G satisfied the Dahlquist criterion (G' < 10^5^ Pa at 1 rad/s), which is essential for robust yet reversible PSA behavior [18].

**Fig. 2:**
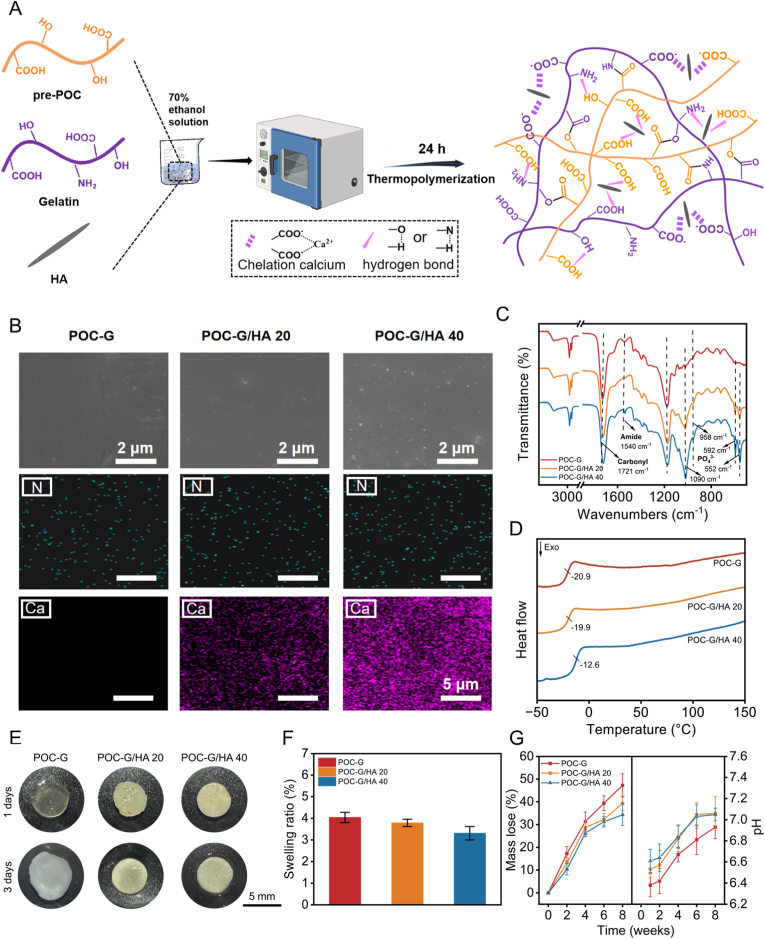
Synthesis and characterization of POC-G/HA bandages. (A) Schematic of the preparation process and structure of the POC-G/HA. (B) SEM and elemental mapping analysis of POC-G. POC-G/HA 20 and POC-G/HA 40. (C) FTIR spectra of POC-G, POC-G/HA 20 and POC-G/HA 40 in the range of 500–4000 cm^−1^. (D) Differential scanning calorimetry (DSC) of the POC-G, POC-G/HA 20 and POC-G/HA 40. The inflection point was defined as the glass transition temperature. (E) Photographs and (F) swelling ratio of patches immersed in PBS for 1 and 3 days (n = 4). (G) Mass loss and pH evolution of the mediators during degradation of the adhesive bandages (n = 4).

To further endow the POC-G adhesive with osteogenic activity, we incorporated HA nanoparticles with well-established osteogenic properties into the polymer phase, thereby obtaining an organic–inorganic composite adhesive. Specifically, HA nanoparticles with mass fractions of 20 wt% (POC-G/HA 20) and 40 wt% (POC-G/HA 40) in proportion to the polymer mass were blended with pre-POC and gelatin in a 70 % ethanol solution to achieve homogeneous distribution of HA within the polymer network (Fig. 2A). After a 24 h thermal polymerization at 70 °C under appropriate conditions for achieving viscoelasticity, followed by mold casting, a milky-white POC-G/HA composite bandage was obtained. Scanning electron microscopy (SEM) coupled with elemental mapping was employed to assess the microscopic dispersion of gelatin and hydroxyapatite (HA) within the POC-G/HA composites. A uniform distribution of nitrogen (N), characteristic component of gelatin, was observed in both POC-G and POC-G/HA groups. Similarly, HA-specific elements calcium (Ca) and phosphorus (P) exhibited homogeneous distribution throughout the composites, with POC-G/HA 40 showing stronger Ca and P signals than POC-G/HA 20 (Fig. 2B). FT-IR further confirmed the successful incorporation of HA (Fig. 2C). Characteristic phosphate (PO_4_^3−^) absorption peaks of HA appeared at 1090 cm^−1^, 958 cm^−1^, 592 cm^−1^ and 552 cm^−1^ [27]. Compared with the amphiphilic POC-G polymer, the POC-G/HA composites displayed reduced intensities at 1720 cm^−1^ (ester C=O stretching) and 1170 cm^−1^ (C–O stretching), suggesting possible chelation between carboxyl groups of POC-G and calcium ions from HA. In addition, the C=O stretching vibration band red-shifted from 1721 cm^−1^ to 1708 cm^−1^, indicative of strong hydrogen-bonding interactions among POC, gelatin, and HA (Fig. 2C). These FTIR observations were consistent with differential scanning calorimetry (DSC) results. The *T*_g_ increased from −20.92 °C to −12.65 °C (Fig. 2D), which can be attributed to enhanced intermolecular interactions between HA and the POC-G network [30,31].

We further investigated the swelling and degradation behavior of the adhesive patches under physiological conditions. The POC-G and POC-G/HA composites exhibited remarkably low swelling ratios with values of 4.04 ± 0.34 % (POC-G), 3.79 ± 0.17 % (POC-G/HA 20), and 3.31 ± 0.31 % (POC-G/HA 40) (Fig. 2E and F). Such limited swelling was attributed to the hydrophobic nature of the POC polymer network. The in vitro degradation of the adhesive patches was subsequently evaluated in PBS, which revealed a progressive mass loss over time. After 8 weeks, the degradation ratios reached 47.22 ± 5.13 % for POC-G, 39.34 ± 3.12 % for POC-G/HA 20, and 34.33 ± 4.78 % for POC-G/HA 40 (Fig. 2G). The slower degradation of HA-containing composites was attributed to the HA-induced increase in crosslinking density, which enhanced the structural stability of the network. It is noteworthy that, due to the intrinsic acidity of POC, the incubation medium of POC-based composites exhibited a markedly reduced pH after one week. Incorporation of HA alleviated this acidity, as evidenced by the more neutral pH values observed in POC-G/HA 20 and POC-G/HA 40 groups (Fig. 2G) [32].

### 3.2 Mechanical properties of the bone adhesive bandage

We investigated the effect of HA nanoparticle concentration on the viscoelastic properties of the bone adhesives using rheological analysis. Oscillatory frequency sweep measurements at room temperature revealed that both pure POC-G polymer and POC-G/HA composites exhibited typical frequency-dependent behavior of polymer melts, with a slightly lower storage modulus (G′) than the loss modulus (G″) (Fig. 2A). The addition of HA nanoparticles significantly enhanced the elasticity of the composites, as evidenced by the notably higher G' = 58.98 ± 2.90 kPa at 1 rad/s for POC-G/HA 40 compared to pure POC-G (G' = 4.2 ± 0.13 kPa at 1 rad/s) (Fig. S5). Importantly, in the low-frequency regime relevant to bonding, all samples exhibited G'' > G′, indicating a liquid-like flow, which is essential for instant adhesion to substrates. Meanwhile, POC-G/HA composites maintained elevated G′ values, confirming strong cohesive strength while preserving characteristic PSA behavior.

**Fig. 3:**
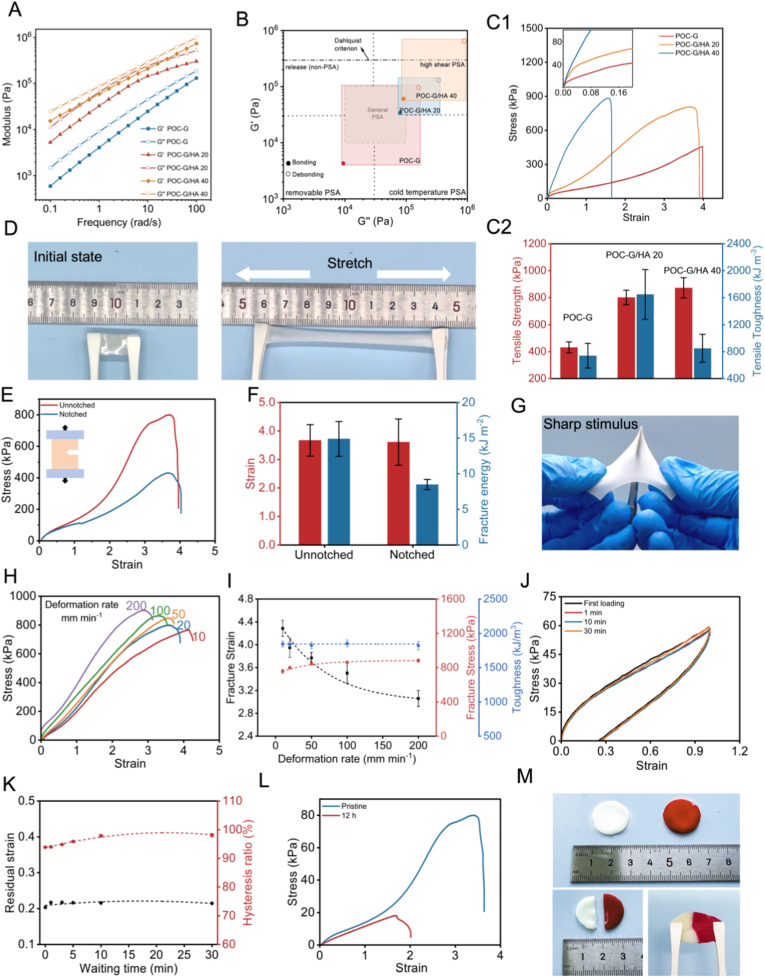
Viscoelasticity and mechanical properties of bone adhesive patches. (A) Oscillatory frequency sweep test showing the viscoelastic behavior the POC-G, POC-G/HA 20, and POC-G/HA 40. (B) Viscoelastic windows of POC-G, POC-G/HA 20, and POC-G/HA 40 based on frequency scanning (Dahlquist criterion, G′<3.0 × 10^5^ Pa). Bonding and debonding frequencies correspond to 1 and 100 rad s^−1^, respectively. (C1) Stress–strain curves, and (C2) tensile strength and tensile toughness of POC-G, POC-G/HA 20, and POC-G/HA 40 (n = 4). (D) Photographs showing the stretch process of POC-G/HA 20. (E) Tensile tests on the notched and unnotched samples of POC-G/HA 20, and the corresponding (F) fracture strain and fracture energy (n = 3). (G) Photograph showing the high toughness of the POC-G/HA 20 composite bandage, which can tolerate the punch of sharp. (H) Stress - strain curves of the POC-G/HA 20 upon tensile loading at different deformation rates (from 10 to 200 mm min^−1^). (I) The fracture strain, fracture stress and toughness of POC-G/HA 20 under different deformation rates (n = 3). (J) Hysteresis curves under different waiting times during the loading and unloading processes of POC-G/HA 20. (K) The residual strain and hysteresis ratio (area ratio of the second hysteresis loop to the first) of POC-G/HA 20 under different waiting times (n = 3). (L) Photographs showing the self-repairing behavior of POC-G/HA 20. The bandage was cut into completely separate pieces, then placed back in a closely attached state for 2 h, and then stretched. The bandage on the right side was coated with red dye. (M) The tensile stress-strain curves of POC-G/HA 20 before self-repair and 2 h after healing.

The viscoelastic window concept, which evaluates dynamic viscoelastic properties at bonding (≈1 rad/s) and debonding (≈100 rad/s) frequencies, was further employed to elucidate performance differences in PSAs [29]. By superimposing each sample's viscoelastic window onto G'/G″ plots at these frequencies, application potential was assessed using the quadrant method (Fig. 3A and B). Both POC-G and POC-G/HA adhesives exhibited storage moduli below the Dahlquist criterion (G'<3 × 10^5^ Pa) at the bonding frequency, confirming their effectiveness as PSAs [33]. Quadrant analysis classified POC-G as a conventional removable PSA. Notably, the incorporation of HA altered the adhesive behavior; POC-G/HA 20 and POC-G/HA 40 showed improved shear resistance, with their viscoelastic windows predominantly occupying the upper-right quadrant, characteristic of high-shear PSAs. This indicates robust cohesion of our POC-G/HA adhesives to resist shear deformation while maintaining structural stability, which is the key for long-term in vivo adhesion integrity [34].

To further characterize the mechanical properties of the bone adhesive bandage, conventional tensile tests were performed. Pure POC-G exhibited a tensile strength of 432.33 ± 41.23 kPa and an elongation at break of 423.45 ± 43.24 % (Fig. 3C, D and S6). In comparison, POC-G/HA composites showed significantly enhanced tensile strengths of 801.46 ± 53.21 kPa and 872.36 ± 75.45 kPa for formulations containing 20 and 40 wt% HA, respectively (Fig. 2C). However, HA incorporation reduced stretchability, and POC-G/HA 40 displayed an elongation at break of 183.67 ± 22.12 %, less than half that of the pure POC-G matrix. Tensile toughness analysis revealed that POC-G/HA 20 achieved 1648.66 ± 367.81 kJ m^−3^, more than twice that of POC-G (741.33 ± 183.41 kJ m^−3^) (Fig. 2E). These findings indicate that multiple reversible interactions within the composite matrix—including hydrogen bonding among polymer components and coordination between Ca^2+^ and POC carboxyl groups—facilitate energy absorption and dissipation through bond rupture and reformation, thereby enhancing toughness. In contrast, excessive incorporation of HA (40 wt%) further increased stiffness but disrupted network continuity due to the presence of solid inorganic fillers, ultimately compromising extensibility.

We further selected the POC-G/HA 20 composite bandage as a representative formulation to investigate notch sensitivity. Pre-introduced notches remained stable during stretching, with negligible crack propagation even under progressive deformation (Fig. 3E). Consistently, the stress–strain curves of notched and unnotched POC-G/HA 20 samples exhibited nearly identical elastic moduli and tensile strains, indicating that the material preserved its load-bearing capacity despite the presence of flaws. Quantitative analysis revealed that while the fracture strain (ε) of notched samples (ε = 3.60 ± 0.55) was comparable to that of unnotched counterparts (ε = 3.67 ± 0.55), the fracture energy (W) was substantially reduced (8.486 ± 0.71 vs. 14.88 ± 2.43 kJ m^−2^) (Fig. 2F). This reduction may be attributed to localized stress concentration at the notch, which promotes premature bond rupture and reduces the total energy dissipated before failure. The notch-insensitive macroscopic behavior was nonetheless supported by abundant reversible sacrificial interactions within the organic–inorganic network—including hydrogen bonding, ionic coordination, and polymer chain entanglement—which dissipated energy during deformation and prevented catastrophic crack propagation. Such energy-dissipating mechanisms allowed the adhesive to retain exceptional toughness, even though the absolute fracture energy decreased in the presence of flaws. To visually demonstrate this toughness, we showed that POC-G/HA 20 bandages can withstand puncture by sharp objects and can be stretched to four times longer of their original length even after twisting (Fig. 2G). These results highlight the ability of POC-G/HA 20 to maintain structural integrity under mechanical challenge and underscore its potential clinical utility in bone fixation.

Unlike permanent covalent crosslinks, the reversible noncovalent interactions (e.g., hydrogen bonding and ionic coordination) in the POC-G/HA network have finite lifetimes and can relax, leading to a strain-rate-dependent mechanical response. To investigate this, we selected POC-G/HA 20 as a representative formulation and performed tensile tests at strain rates ranging from 10 to 200 mm min^−1^ (Fig. 3H). As shown in Fig. 3I, increasing the strain rate resulted in higher fracture stress but reduced fracture strain. This behavior was attributed to the limited relaxation of reversible bonds under rapid loading, which enhances strength but restricts extensibility, whereas slower loading allows bond dissociation and reformation, thereby increasing ductility at the expense of maximum stress. Meanwhile, at different stretching rates, the adhesive bandages exhibited excellent resilience retention, indicating the robustness and dynamic adaptability of their network structure. The POC-G/HA composites also exhibited excellent fatigue resistance with stable self-recovery under cyclic deformation. As shown in Fig. 2J, the first loading–unloading cycle displayed distinct yielding and pronounced hysteresis, indicative of substantial energy dissipation. After a short relaxation period (∼1 min), the stress–strain curve largely recovered toward the original trajectory. With extended relaxation time, the recovery improved further, and subsequent loading–unloading cycles showed nearly overlapping curves, while a residual strain of ∼20 % was consistently observed (Fig. 3J and K). Such residual strain is characteristic of reversible crosslinked networks, reflecting their high capacity for bond breakage and reformation and underscoring the material's ability to dissipate energy during deformation without irreversible chain sliding.

In addition, POC-G/HA composites demonstrated remarkable self-healing behavior. To verify this property, composite bandages were cut into two pieces and brought into direct contact at freshly cut interfaces. Spontaneous interfacial adhesion was observed, indicating the ability of the composites to re-establish cohesive bonding without external stimuli (Fig. 3L). Self-healing was further evaluated by tensile testing after a defined resting period of approximately 2 h (Fig. 3M). The stress–strain curves of pristine and self-healed samples exhibited comparable elastic moduli, with a modulus recovery efficiency of ∼95 %, confirming that the reconstructed network effectively restored stiffness. However, the fracture strain decreased from 375 % (pristine) to 200 % (healed), indicating a loss of extensibility. This reduction was attributed to the incomplete recovery of long-range polymer chain alignment, even though reversible bonds such as hydrogen bonding, ionic coordination, and chain entanglement can reform at the damaged interfaces. Together, these findings demonstrate that POC-G/HA composites possess robust self-healing ability, restoring stiffness and interfacial integrity, while highlighting the inherent trade-off between stiffness recovery and reduced deformability—a common feature of dynamic reversible polymer networks.

### 3.3 The adhesion behavior of the bone adhesive bandage

**Fig. 4:**
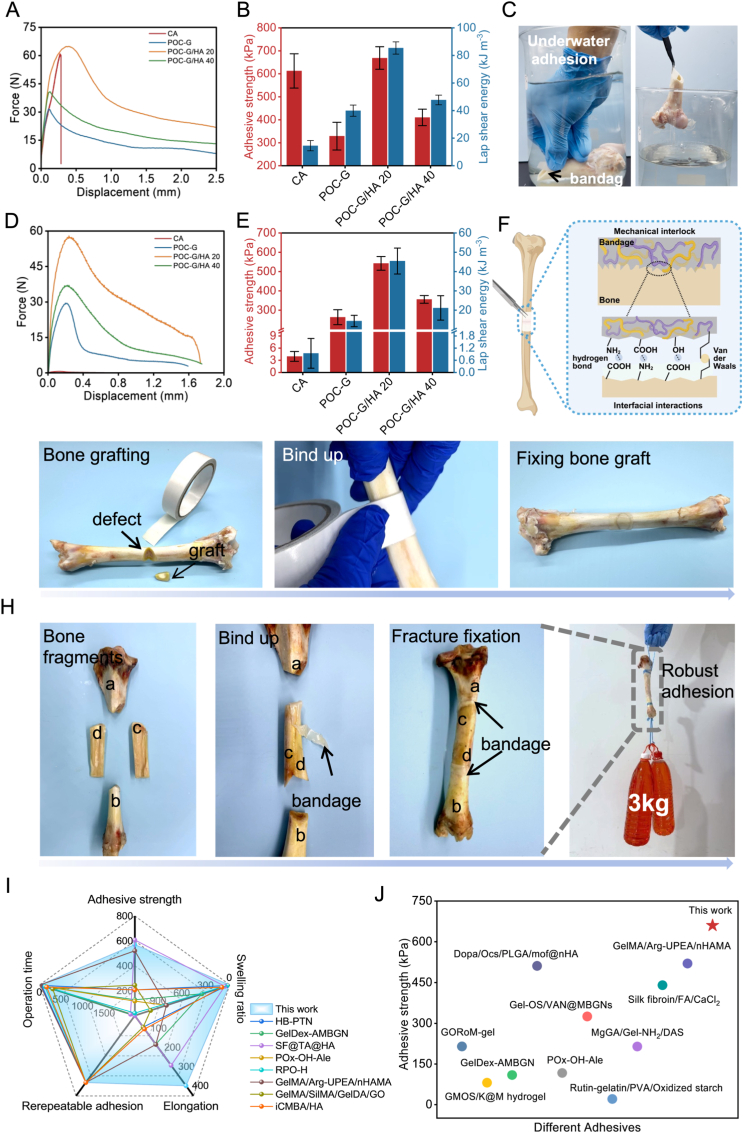
Characterization of the adhesive properties of bone adhesive patches. (A) Representative Lap-shear curves and (B) adhesive strength of CA, POC-G, POC-G/HA 20, and POC-G/HA 40 bandages in air (n = 4). (C) Photographs of POC-G/HA 20 bandages adhering to bones underwater. (D) Representative Lap-shear curves and (E) adhesive strength of CA, POC-G, POC-G/HA 20, and POC-G/HA 40 in PBS (n = 4). (F) Schematic diagram of the adhesion mechanism of bone adhesive bandages. A series of photographs showing POC-G/HA 20 fixed (G) bone graft and (H) bone fragments. Binding with bandages achieves rapid stabilization of the graft and bone fragments. (I) Radar chart showing the adhesive and mechanical properties of POC-G/HA 20 and the reported adhesives across key dimensions: adhesive strength, swelling ratio, elongation break, repeatable adhesion, and operation time. (J) Comparison of the adhesive strength and operation time of POC-G/HA 20 adhesive with other reported adhesives on bovine bone.

POC-G/HA demonstrated remarkable adhesion to diverse substrates through multiple reversible interactions, enabling rapid and stable bonding to bone tissue even in aqueous environments. The adhesion strength of POC-G/HA to bone was evaluated under both wet and dry conditions using lap shear testing, with comparisons made against POC-G/HA composites of varying HA contents and commercial cyanoacrylate (CA, Compont®, China). Maximum adhesion strength and fracture energy during detachment were recorded to comprehensively assess adhesive performance. Typical lap shear stress–strain curves revealed two distinct regimes: an initial sharp increase in stress with deformation, followed by a gradual decline until adhesive failure. POC-G/HA exhibited robust underwater bone adhesion, with lap shear strengths significantly exceeding those of commercial cyanoacrylate (CA) adhesives. Specifically, POC-G/HA 20 and POC-G/HA 40 achieved adhesion strengths of 668.67 ± 49.08 kPa and 410.3 ± 35.8 kPa, respectively, compared to 612.59 ± 74.68 kPa for CA (Fig. 4A and B). In humid environments, the performance of CA deteriorated drastically because its monomers undergo rapid anionic polymerization upon contact with water, resulting in brittle fracture and poor interfacial bonding. This was reflected in its markedly reduced adhesion strength (CA, 3.95 ± 1.21 kPa), whereas POC-G/HA 20 maintained strong bonding capacity (570.43 ± 21.82 kPa) under the same conditions (Fig. 4C–E), confirming the aqueous stability of the composite adhesive. Although the adhesive strength of POC-G/HA 40 is slightly lower than that of POC-G/HA 20, it still meets the currently accepted minimum requirement of 200 kPa [35], and surpasses the results reported in some previous studies [[36], [37], [38]]. These comparisons further support the adequacy and reliability of POC-G/HA 40 for bone adhesion.

Fracture energy measurements further highlighted the intrinsic differences between the two systems. POC-G/HA composites exhibited significantly higher fracture energies (POC-G/HA 20, 45.46 ± 6.72 kJ m^−3^; POC-G/HA 40, 21.15 ± 6.32 kJ m^−3^) compared to CA (0.94 ± 0.73 kJ m^−3^) (Fig. 4E). This enhancement can be attributed to the viscoelastic nature of the POC-G/HA network, where multiple reversible interactions (hydrogen bonding, ionic coordination, and physical entanglement) enable efficient energy dissipation during debonding. In contrast, CA fractures in a brittle manner upon stress application, leading to insufficient energy absorption at the interface and consequently lower fracture energy. Even without considering the interruption of water molecules for CA adhesion reactions, CA adhesives typically exhibited brittle fracture behavior with rapid stress decline post-peak. In contrast, POC-G/HA adhesives demonstrated more robust adhesiveness with progressive stress reduction after reaching maximum strength, which can avoid the adhesion from unwanted sudden mechanical and structural failure (Fig. S7). The strong adhesion of POC - G/HA can be ascribed to the following aspects: (i) The POC segments contain abundant hydrophobic moieties derived from 1,8-octanediol and ester groups. These hydrophobic domains effectively disrupt the hydration layer on the bone surface, thereby enabling intimate contact between the adhesive and wet bone tissue. Once water is excluded from the interface, multiple hydrogen bonds are formed between carboxyl, hydroxyl, and amino groups in the composite and the functional groups present on bone tissue, (ii) the composite is free of residual solvent in its final state. This creates a high-density polymer chain network at the adhesive surface, offering more available interaction sites and further enhancing interfacial bonding, (iii) the viscoelasticity of the composite also enables mechanical interlocking with the microscopic surface rough topography of the fracture bone, improving resistance to shear stress during debonding. (Fig. 4F).

Other than exhibiting strong adhesion strength, POC-G/HA composites also demonstrated repeatable adhesive performance, as reflected by the maintained adhesion strength values after 20 consecutive adhesion–detachment cycles without significant performance loss (Fig. S8). Furthermore, the composites retained long-term adhesion to diverse substrates regardless of their hydrophilic or hydrophobic nature, including glass, steel, rubber, and plastics (Fig. S9), as well as various wet tissues such as skin, heart, adipose, bone, and stomach (Fig. S10). Importantly, POC-G/HA maintained stable underwater adhesion, as evidenced by robust bonding to bone slices even after immersion in water for more than five days (Fig. S11), confirming its durability under physiologically relevant conditions. To further validate the bone adhesion capability of our composite, we established a comminuted defect model in a sheep tibia and adhered the bone fragments using the optimized POC-G/HA 20 bandage (Fig. 4G). Notably, POC-G/HA 20 enabled rapid and stable integration of fractured bone pieces within 1 min. In a more challenging model involving four segmental and comminuted fractures, POC-G/HA 20 also achieved complete adhesion of the separated bone segments, reconstructing the bone into a continuous structure. The repaired bone was able to lift a 3-kg weight without detachment, demonstrating that the optimized composite formulation provided instant structural integrity and fixation (Fig. 4H). By contrast, POC-G/HA 40, although capable of bonding bone segments, exhibited lower flexibility and less favorable handling properties, underscoring the superior balance of adhesion and toughness achieved with the 20 wt% HA formulation.

To benchmark the performance of our POC-G/HA adhesives, we compared them with previously reported bone adhesives across multiple functional parameters (Fig. 4I). The radar plot clearly highlights that the POC-G/HA composites outperform most existing hydrogel- or polymer-based adhesives in terms of adhesion strength, swelling resistance, elongation, repeatable adhesion, and operation time. Specifically, the POC-G/HA 20 bandage achieved an adhesion strength of 570.43 ± 21.82 kPa on wet bone tissues, while maintaining excellent elongation at break (up to 423.45 %). This combination of strong adhesion and high stretchability is rarely achieved in previously reported systems, where adhesives typically sacrifice extensibility in order to gain strength. Moreover, the low swelling ratio (<4 %) of the POC-G/HA network ensures dimensional stability in aqueous physiological environments, preventing premature debonding or dilution of adhesive properties—a limitation often encountered in hydrogel-based formulations. The adhesive bandage also exhibited stable performance over 20 consecutive adhesion–detachment cycles, reflecting its robust repeatability and reusability. In addition, its operation time (∼1 min to achieve stable fixation) is substantially shorter than that of most reported hydrogel adhesives, which often require in-situ curing or complex handling steps, thereby demonstrating clear clinical advantages.

Fig. 4J further provides a direct comparison of adhesion strength among different bone adhesives reported in the literature. While most hydrogel-based bone adhesives exhibit adhesion strengths below 300 kPa, and commercial systems such as Gel-OS/VAN@MBGNS or Rutin–gelatin/PVA/oxidized starch hydrogels remain in the range of ∼150–350 kPa, our POC-G/HA 20 formulation achieved ∼570 kPa. This value not only surpasses the majority of emerging hydrogel adhesives but also approaches or exceeds the performance of clinical bio-glues, such as cyanoacrylate and PMMA bone cement. Importantly, unlike these clinically used adhesives, which rely on rapid in-situ curing but suffer from brittleness and poor degradation profiles, the POC-G/HA composites combine high adhesive strength with biodegradability, toughness, and osteogenic activity. Taken together, the comprehensive comparison in Fig. 4I and J underscores the superiority of the POC-G/HA adhesive system. Its unique combination of strong and repeatable adhesion, low swelling, high elongation, short operation time, and outstanding adhesion strength positions it as a highly competitive candidate for clinical translation in bone fixation and regeneration.

### 3.4 Biocompatibility and biodegradability of the POC-G/HA adhesive

**Fig. 5:**
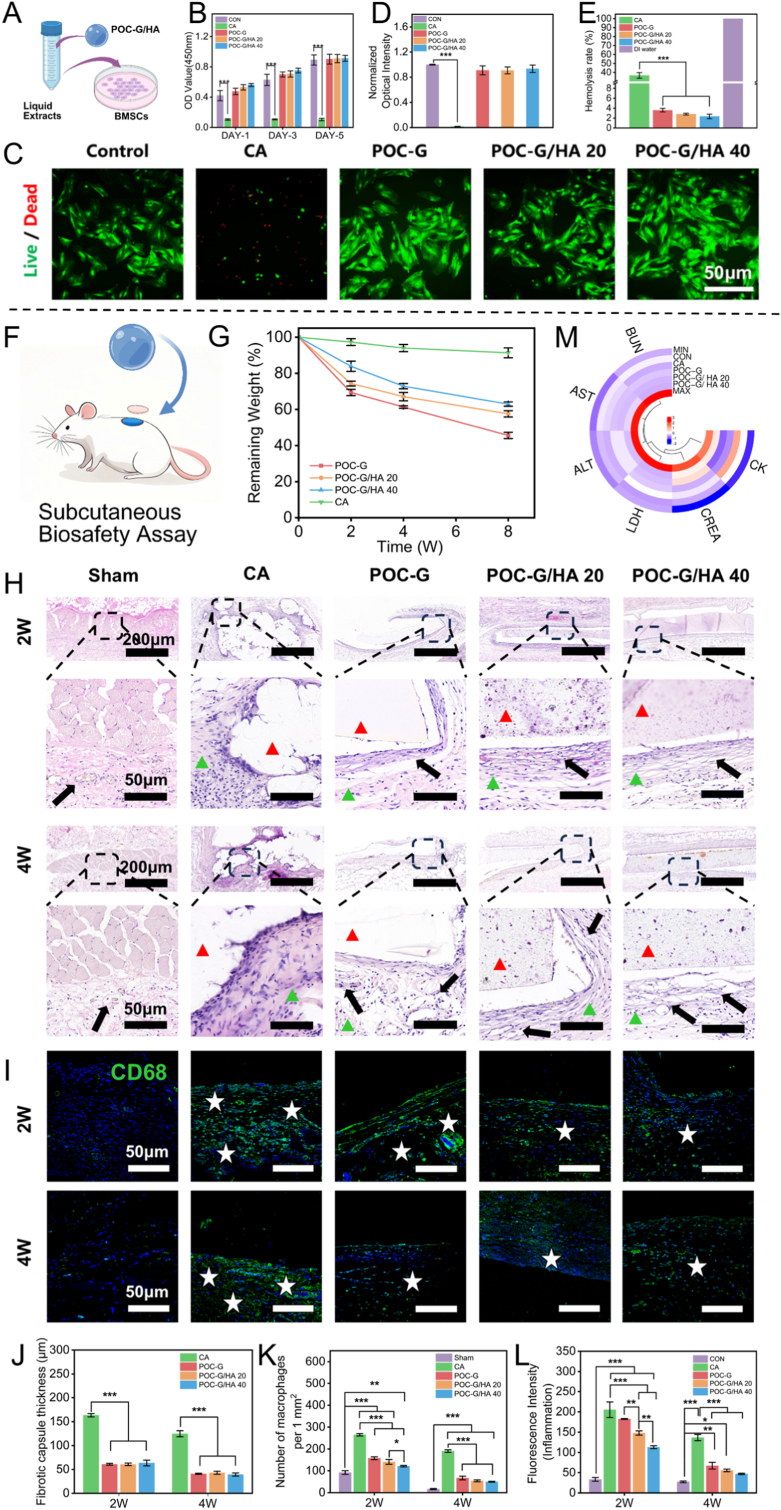
Biocompatibility and biodegradability of POC-G/HA bone adhesives. (A) Flowchart of culturing rBMSCs using liquid extracts prepared with POC-G/HA bone adhesives in comparison to CA bio-glue. (B) Viability of rBMSCs treated with CA, POC-G, POC-G/HA 20, POC-G/HA 40 or the control for 1, 3, and 5 days (n = 3, CCK-8 assay). (C) Representative images of the Live/Dead staining of rBMSCs after incubation with CA, POC-G, POC-G/HA 20, POC-G/HA 40 or the control for 3days. (D) Quantitative analysis of Live/Dead staining fluorescence intensity in rBMSCs. (E)Hemocompatibility evaluation of CA, POC-G, POC-G/HA 20 and POC-G/HA 40 at 37 °C for 4 h (n = 3). (F) Surgical schematic illustration of the subcutaneous implantation model on the backs of SD rats (n = 3). (G) Remaining weights of the CA, POC-G, POC-G/HA 20, POC-G/HA 40 tokens after implantation for 2, 4, and 8 weeks (n = 6). (H) H&E staining of the implantation sites after implantation for 2 and 4 weeks (red triangle: implant material; green triangle: inflammatory cell infiltration in the tissue; black arrow: neovascularization). (I) CD68 immunofluorescence staining of the implantation sites after implantation for 2 and 4 weeks (white star: macrophages). Quantification of fibrous capsule thickness (J), inflammatory cell infiltration (K) and CD68 fluorescence intensity (L) at the implantation site (n = 3). (M) Heatmap of the levels of routine blood parameters (ALT, AST, BUN, CK, CREA, and LDH) in SD rats after treatment for 4 weeks (n = 3). Statistical significance: ∗p < 0.05, ∗∗p < 0.001, ∗∗∗p < 0.001 (ANOVA). (For interpretation of the references to color in this figure legend, the reader is referred to the Web version of this article.)

To further assess the biocompatibility of the POC-G/HA bone adhesives, we conducted in vitro cell culture experiments using a commercially available CA bio-glue as a comparator. In accordance with ISO 10993–5:2009 (E), liquid extracts of the POC-G/HA adhesive and CA bio-adhesive were prepared in culture medium to facilitate cellular morphological observation and subsequent experimental investigations [39,40]. Rat bone marrow mesenchymal stem cells (rBMSCs) were then cultured in these extracts (Fig. 5A) and divided into three groups, namely a blank control (standard culture medium only), CA, and POC-G/HA (extract-treated). Cell proliferation was quantified using a Cell Counting Kit-8 (CCK-8). As shown in Fig. 5B, POC-G, POC-G/HA 20, and POC-G/HA 40 groups exhibited no significant difference in cell viability compared with the control at 1, 3, and 5 days, whereas CA markedly reduced optical density, indicating severe inhibition of cell survival. A subsequent live/dead assay further confirmed these findings. After 3 days, only a few dead cells (red) were observed in the control and POC-G/HA groups, with no statistically significant difference in viability, while the CA group exhibited a cell death rate of 98.4 % (Fig. 5C and D). In addition, hemolysis assays revealed negligible hemolytic activity in the POC-G/HA group, in stark contrast to the significant hemolysis induced by CA (Fig. 5E–S13). Taken together, these results demonstrate the minimal cytotoxicity and superior biosafety of POC-G/HA bone adhesives compared with the clinically used CA bio-glue.

To further evaluate the in vivo biocompatibility and biodegradability of the POC-G/HA adhesive bandage, a subcutaneous implantation model was established using Sprague Dawley (SD) rats (Fig. 5F). Rats were randomly divided into five groups (Sham, CA, POC-G, POC-G/HA 20, and POC-G/HA 40). The samples were fabricated into cylindrical discs with a diameter of 1 cm and a height of 1 mm, and subsequently implanted subcutaneously in the dorsal region of the rats. At 2, 4, and 8 weeks post-implantation, the implantation sites were reopened and the residual dry weights of the retrieved discs were measured to determine the degradation profiles. After 8 weeks, CA implants exhibited minimal degradation with a mass loss of only 8.77 ± 2.76 wt%, whereas the POC-based composites displayed markedly higher degradation. Specifically, mass loss was 55.42 ± 1.72 % for POC-G, 42.46 ± 1.73 % for POC-G/HA 20, and 37.06 ± 1.21 % for POC-G/HA 40 (Fig. 5G). These results clearly indicate that incorporation of HA nanoparticles substantially enhanced the structural stability of the polymer networks, thereby retarding degradation relative to pure POC-G.

An ideal bone adhesive should undergo gradual degradation synchronized with the natural process of fracture healing, thereby eliminating the need for secondary removal surgery. Importantly, the degradation kinetics must strike a balance between two unfavorable extremes, namely (i) excessively rapid degradation, which leads to loss of mechanical integrity, impaired adhesion stability, and diminished functional efficacy, and (ii) overly slow degradation, which hinders osteointegration by obstructing new bone ingrowth and inducing chronic inflammation, ultimately prolonging recovery [41]. Our POC-G/HA adhesive bandage effectively addresses these challenges by maintaining stable interfacial adhesion without penetrating bone fragment gaps under physiological conditions, while simultaneously achieving sustained degradation within 8 weeks. This degradation profile creates an optimal microenvironment for bone trauma repair. Following material implantation, the host typically recruits macrophages along with non-inflammatory cells to initiate the inflammatory cascade, a process that eventually culminates in the formation of a fibrous capsule [42].

To assess the host tissue responses to POC-G/HA adhesives, fibrous encapsulation and cellular infiltration surrounding the implants were examined by H&E staining (Fig. 5H). At 2 weeks post-implantation, the fibrous capsule thickness for CA was 163.37 ± 3.44 μm, which was significantly greater than that of POC-G (60.99 ± 1.80 μm), POC-G/HA 20 (60.78 ± 2.59 μm), and POC-G/HA 40 (63.57 ± 5.98 μm) (p < 0.001). By week 4, capsule thickness in the POC-G, POC-G/HA 20, and POC-G/HA 40 groups further decreased to 40.71 ± 1.19 μm, 42.94 ± 3.13 μm, and 39.31 ± 3.24 μm, respectively, whereas CA implants maintained a markedly thicker capsule of 124.68 ± 6.30 μm (p < 0.001) (Fig. 5J). Macrophage accumulation at implantation sites was also quantitatively analyzed. At 2 weeks, the Sham group exhibited a macrophage density of 91.78 ± 8.43 cells mm^−2^, while CA implants induced a significantly higher density of 264.78 ± 5.29 cells mm^−2^. In contrast, POC-G (157.89 ± 6.38 cells mm^−2^), POC-G/HA 20 (141.11 ± 10.86 cells mm^−2^), and POC-G/HA 40 (121.56 ± 3.40 cells mm^−2^) all showed substantially lower macrophage infiltration compared with CA (p < 0.001), indicating a milder inflammatory response during the early healing phase. At 4 weeks, macrophage density declined in all groups, reaching 16.44 ± 2.47 cells mm^−2^ in Sham, 66.89 ± 8.35 cells mm^−2^ in POC-G, 54.00 ± 4.25 cells/mm^2^ in POC-G/HA 20, and 49.89 ± 2.04 cells mm^−2^ in POC-G/HA 40. However, CA implants still displayed persistently high macrophage density at 190.44 ± 5.72 cells mm^−2^ (p < 0.001), suggesting stronger foreign body reactions compared with POC-G/HA composites (Fig. 5K). Furthermore, extensive neovascularization was observed around the implantation sites of POC-G/HA composites (Fig. 5H, black arrows), whereas CA implants showed negligible vascular regeneration, further indicating impaired inflammatory regulation.

Further postoperative immunofluorescence analysis demonstrated markedly reduced CD68^+^ macrophage infiltration in the POC-G, POC-G/HA 20, and POC-G/HA 40 groups compared with the CA group, which exhibited substantial inflammatory cell accumulation (Fig. 5I). Importantly, a dose-dependent effect of HA was observed, with POC-G/HA 40 showing significantly lower CD68^+^ fluorescence intensity (112.96 ± 3.62 a.u.) than POC-G/HA 20 (147.92 ± 5.28 a.u., p < 0.001) at 2 weeks post-implantation. Relative to the Sham group (33.37 ± 4.56 a.u.), both POC-G (181.44 ± 1.03 a.u.) and CA (205.18 ± 19.18 a.u.) displayed robust inflammatory responses, whereas the POC-G/HA composites exhibited significantly attenuated signals (p < 0.001), highlighting the anti-inflammatory contribution of HA. By 4 weeks, fluorescence intensities of the POC-G/HA composites declined markedly to 55.71 ± 3.30 a.u. (POC-G/HA 20) and 46.91 ± 1.63 a.u. (POC-G/HA 40), approaching values comparable to Sham (27.33 ± 2.06 a.u.). In contrast, CA implants continued to elicit persistent inflammatory responses, with fluorescence intensity remaining at 136.70 ± 7.63 a.u. (p < 0.001) (Fig. 5L). Collectively, these findings confirm that POC-G/HA adhesives substantially mitigate immune activation compared with the clinically used CA bio-glue. Notably, although citrate release during POC-G degradation could transiently reduce local pH, incorporation of HA effectively neutralized acidity and prevented severe inflammatory reactions. This buffering effect underlies the superior biocompatibility observed in POC-G/HA 40 compared with POC-G/HA 20 and pure POC-G (Fig. 2G).

Further histological examination by H&E staining of major organs (heart, liver, spleen, lungs, and kidneys) at 4 weeks post-implantation was performed to assess potential systemic side effects and long-term biocompatibility (Fig. S14). No pathological alterations were observed in the POC-G/HA-treated groups. At the same time point, blood samples were collected for serum biochemical analysis, and the results demonstrated that POC-G/HA adhesives did not induce significant changes in routine hematological or biochemical parameters. Indicators of cardiac, hepatic, and renal function, including alanine transaminase (ALT), aspartate aminotransferase (AST), blood urea nitrogen (BUN), creatinine (CRE), creatine kinase (CK), and lactate dehydrogenase (LDH), remained within normal ranges (Fig. 5M). Collectively, these findings confirm the excellent biocompatibility of POC-G/HA adhesives and highlight their potential for long-term clinical applications. Moreover, it is noteworthy that in the subcutaneous implantation experiment, the POC-G/HA bone adhesive demonstrated excellent inflammatory modulation capabilities and promoted angiogenesis in the surrounding tissue, which may represent a key factor in facilitating the regeneration and repair of bone tissue [43,44].

### 3.5 In vitro investigation of the osteogenic capacity of POC-G/HA adhesive

**Fig. 6:**
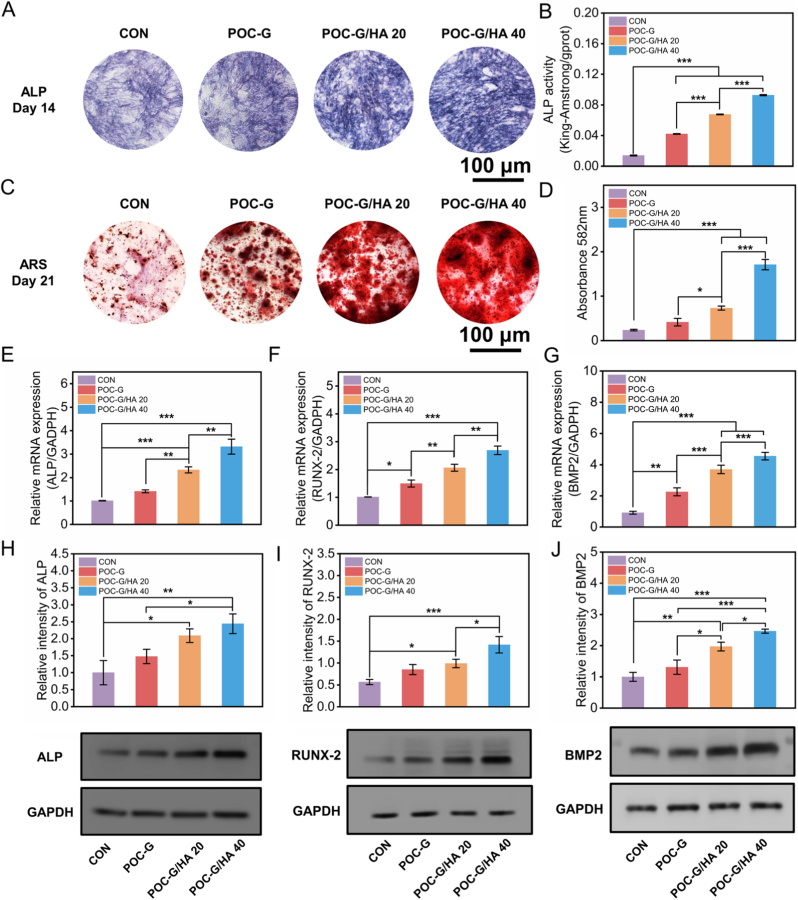
In vitro osteogenic capacity of POC-G/HA bone adhesives. (A) ALP staining and (C) ARS staining of rBMSCs cultured with POC-G/HA adhesives. Quantitative analysis of ALP activity (B) and ARS staining (D). RT-qPCR results of ALP (E), Runx-2 (F), and BMP2 (G) for detecting relative osteogenic genes expression after 14 days incubation. Results were normalized with GAPDH. (n = 3). Western blot analysis of ALP (H), Runx-2 (I) and BMP2 (J) for detecting osteogenic-related proteins expression (n = 3). Statistical significance: ∗p < 0.05, ∗∗p < 0.001, ∗∗∗p < 0.001 (ANOVA).

The critical function of bone adhesives lies in their capacity to promote bone tissue regeneration, underscoring the importance of enhancing osteogenesis for accelerated fracture healing [45]. To evaluate the in vitro osteogenic potential of POC-G/HA adhesives, rBMSCs were cultured in osteogenic induction media preconditioned with different formulations (POC-G, POC-G/HA 20, POC-G/HA 40) for 24 h. Alkaline phosphatase (ALP) activity, an early osteogenic marker [46], was examined at 14 days. ALP staining revealed significantly stronger signals in the POC-G/HA 40, POC-G/HA 20, and POC-G groups compared with the control (no material treatment, p < 0.05), with POC-G/HA 40 exhibiting the highest ALP intensity (Fig. 6A, B and G). Calcium phosphate (CaP) deposition was further evaluated by alizarin red staining at 21 days. Cells treated with POC-G, POC-G/HA 20, or POC-G/HA 40 exhibited dense, red-stained calcified nodules, in sharp contrast to the sparse CaP deposition in the control group (Fig. 6C and D). This enhanced osteogenic differentiation is likely attributable to the presence of HA, a well-established osteoinductive bioceramic. Consistently, simulated body fluid (SBF) immersion experiments confirmed sustained mineralization on pure POC-G composites, as evidenced by increased calcium content. The mineralization effect was further enhanced with the incorporation of HA, demonstrating an HA content-dependent relationship (Fig. S15). To gain molecular insights into this osteogenic efficacy, RT-PCR analysis of key osteogenic genes (BMP-2, Runx-2, and ALP) was performed at day 14. All POC-G-based materials significantly upregulated osteogenic gene expression, with POC-G/HA 40 inducing the highest levels (p < 0.01 vs. control, Fig. 6E–G). Specifically, compared to the blank control group, the relative expression levels of ALP in the POC-G, POC-G/HA 20, and POC-G/HA 40 groups increased by 40.25 ± 6.11 %, 130.52 ± 12.80 %, and 228.52 ± 31.52 %, respectively. Similarly, the expression of Runx-2 increased by 47.98 ± 12.46 %, 104.01 ± 12.24 %, and 165.52 ± 15.11 %, respectively, while BMP-2 expression increased by 147.07 ± 28.10 %, 304.49 ± 29.32 %, and 396.94 ± 25.78 %, respectively. Finally, Western blot analysis corroborated these results, demonstrating HA concentration–dependent upregulation of Runx-2 and BMP-2 protein expression in the POC-G/HA groups (Fig. 6H–J).

The remarkable osteogenic capacity of POC-G/HA composite adhesives is likely attributable to the synergistic effects of hydroxyapatite (HA) and citrate within the composite formulations. HA is well recognized for its ability to adsorb extracellular matrix proteins, such as fibronectin, thereby enhancing osteoblast adhesion and proliferation [4,47,48]. In addition, HA modulates the local microenvironment by releasing calcium (Ca^2+^) and phosphate (PO_4_^3−^) ions. Calcium influx activates calcium-sensing receptors (CaSR), which subsequently trigger the expression of osteogenic markers [31,49], whereas phosphate ions suppress osteoclast activity, maintaining a balanced bone formation–resorption process [50]. Citrate, incorporated through the POC-G backbone, represents another osteoinductive component. Transported via SLC13a5, citrate modulates oxidative phosphorylation during the early stages of mesenchymal stem cell differentiation, elevates cellular energy status, and thereby supports and promotes osteogenic differentiation to meet the high metabolic demands of the bone formation process [21,51,52]. Additionally, Xu et al. demonstrated that citrate, glutamine, and magnesium synergistically activate sustained energy metabolism during osteogenesis through calcium/calmodulin-dependent protein kinase kinase 2 (CaMKK2) and protein kinase B (Akt)-dependent signaling, further confirming the role of citrate in promoting osteogenesis [39]. These processes elevate ATP production, upregulate key osteogenic genes (Runx2, ALP, BMP2), and enhance ALP activity with concurrent calcium deposition in differentiated rBMSCs [23,39]. Consistently, POC-G alone increased ALP expression as evidenced by both PCR and Western blot, indicating that the metabolic activity of rBMSCs was effectively stimulated [53]. Moreover, although degradation of POC can generate a mildly acidic microenvironment, the incorporation of HA mitigates citrate-induced acidity by releasing OH^−^ during its own degradation (Fig. 2G). This buffering effect explains the superior bioactivity of POC-G/HA 40 compared with lower-HA formulations. Collectively, these findings suggest that POC-G and HA act synergistically to promote osteogenesis through complementary biochemical and metabolic pathways.

### 3.6 In vitro demonstration of POC-G/HA-mediated macrophage polarization and angiogenesis

It is well established that bone regeneration and repair constitute a complex process involving not only the osteogenic differentiation of stem cells but also close interactions with immune regulation. The study by Xing et al. confirmed that limiting the excessive activation of the NLRP3 inflammasome in bone marrow-derived macrophages can promote the osteogenic differentiation of BMSCs [54]. The concept of “promoting osteogenesis by modulating the local pathological microenvironment” underscores the importance of manipulating immune cells to establish an osteoimmunological milieu conducive to bone reconstruction [43,55,56]. This property plays a critical role in determining the suitability of biomaterials for bone tissue engineering applications. During acute injury or foreign body response, inflammatory cells (predominantly macrophages) undergo rapid recruitment to initiate inflammation. Initially polarized toward a pro-inflammatory M1 phenotype for pathogen clearance [57,58], macrophages subsequently transit to an anti-inflammatory M2 phenotype that mediates tissue repair. As demonstrated by Xie et al., POCG can suppress acute inflammation by downregulating the expression of pro-inflammatory cytokines (tumor necrosis factor-α (Tnf-α), interleukin-1β (IL-1β), and interleukin-6 (IL-6)) and modulating macrophage polarization toward an anti-inflammatory (M2) phenotype [59]. This effect is likely attributable to the gradual release of citrate during the degradation of POC-G, which inhibits glycolysis-related enzymes, sustains metabolic flux through the tricarboxylic acid (TCA) cycle, and reprograms macrophage metabolism toward oxidative phosphorylation—a hallmark of M2 polarization [21,60]. Exosomes from M2 macrophages can activate the Wnt/β-catenin signaling pathway to promote the osteogenic differentiation of BMSCs, as demonstrated in the study by Zhao et al. [61].

**Fig. 7:**
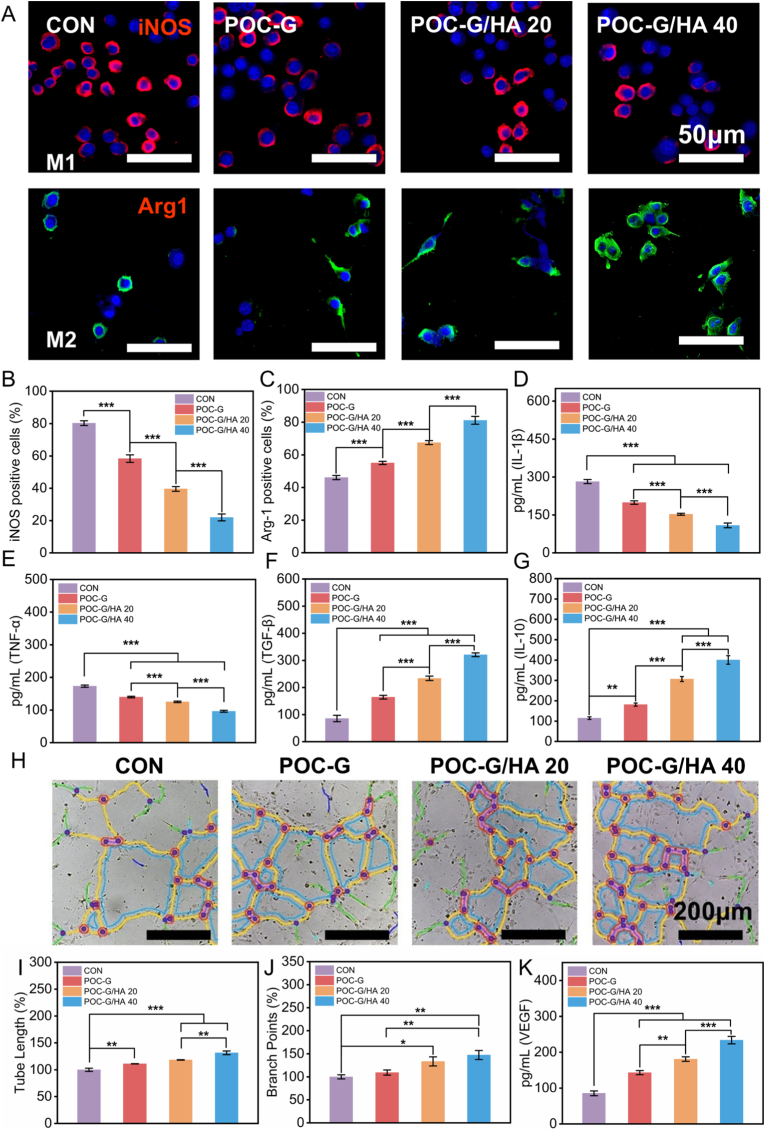
The effect of POC-G/HA bone adhesive on angiogenesis promotion and macrophage polarization in vitro. (A) Immunofluorescence images of RAW264.7 cells induced to differentiate by LPS (100 ng/ml) for 24 h and then cultured with POC-G/HA bone adhesive for 3 days (M1 macrophages: red; M2 macrophages: green). Quantitative analysis of the proportion of iNOS (B, representing M1 macrophages) and Arg1 (C, representing M2 macrophages) positive cells in immunofluorescence staining. ELISA detection of IL-1β (D) and TNF-α (E) secreted by M1 macrophages (n = 3). ELISA detection of IL-10 (F) and TGF-β (G) secreted by M2 macrophages (n = 3). (H)Images of tube formation by HUVECs co-cultured with POC-G/HA for 24 h. Quantification of tube length (I) and branching points of HUVECs (C). (J) ELISA detection of VEGF secreted by HUVECs (n = 3). Statistical significance: ∗p < 0.05, ∗∗p < 0.001, ∗∗∗p < 0.001 (ANOVA).

Thus, we assessed the immunomodulatory effects of POC-G/HA composites on osteogenic differentiation using macrophage cell line RAW264.7, which revealed HA content-dependent macrophage polarization behavior. The results indicate that POC-G accelerates the transition of macrophage polarization from the pro-inflammatory to the anti-inflammatory phenotype. Furthermore, the increased incorporation of HA effectively neutralized the acidity induced by POC-G degradation, establishing a favorable microenvironment for cell viability, which further amplifies the immunoregulatory function of POC-G and reveals an HA content-dependent macrophage polarization behavior. Specifically, POC-G/HA 40 maximally reduced M1 markers (iNOS^+^/CD68^+^) while elevating M2 markers (Arg1^+^/CD68^+^) as compared to that of POC-G/HA 20 and POC-G (Fig. 7A–C). Further ELISA assay confirmed significant reduction in pro-inflammatory cytokines (IL-1β, TNF-α) from M1 macrophages and increased anti-inflammatory cytokines (TGF-β, IL-10) from M2 macrophages for macrophages cultured with POC-G/HA (Fig. 7D–G). Critically, neovascularization recruited more inflammatory cells to the defect site, where POC-G/HA established an early-stage immunoregulatory niche environment (Fig. 4H) [[62], [63], [64], [65]]. This spatiotemporally controlled inflammatory response facilitated angiogenic initiation and cellular preparation, synergistically supporting bone regeneration [63,66,67].

In subcutaneous material implantation experiments in SD rats, we observed extensive neovascularization surrounding the POC-G/HA adhesive implantation site (Fig. 5H). Gelatin, as a product of partial hydrolysis of collagen, retains a significant number of arginine-glycine-aspartic acid (RGD) sequences [68]. These RGD sequences bind to integrin receptors on the surface of endothelial cells and trigger intracellular signaling pathways, thereby promoting cell adhesion, survival, proliferation, and migration, which are closely associated with angiogenesis [69,70]. Beyond immunomodulatory functions, the study by Xie et al. additionally demonstrated that POCG can enhance VEGF and CD31 expression, promote endothelial cell vascularization and accelerate wound repair [59]. Furthermore, previous evidences have demonstrated that external citrate supplementation can activate the PI3K/Akt signaling pathway of human umbilical vein endothelial cells (HUVECs) to enhance the secretion of angiogenic growth factors such as vascular endothelial growth factor (VEGF) and fibrosis growth factor (FGF) [71]. Due to the degradation products of our POC-based composites can also generated more citrate and gelatin at the defective sites, we speculated our POC-G/HA composites may also possess angiogenic capacity. As the primary regulator of blood vessel growth, VEGF is essential for angiogenesis-osteogenesis coupling during skeletal development and post-traumatic repair, promoting endothelial migration/proliferation while modulating osteogenic factors [44]. To this end, the culture medium was incubated with POC-G/HA adhesive for 24 h to prepare liquid extracts, which were then used for HUVEC culture with experimental groups divided into a blank control group (standard culture medium only) and a POC-G/HA group (liquid extract-treated). Subsequently, the pro-angiogenic capacity of POC-G/HA was evaluated through progressive vascular cells migration and closed-loop formation assays, demonstrating its ability to promote angiogenesis [72]. Remarkably, POC-G/HA 40 significantly increased vessel length, branching points (Fig. 7H–J) and wound healing rates (Fig. S16) versus controls. Further ELISA assay confirmed that the POC-G/HA adhesive significantly enhanced VEGF secretion in HUVECs compared to the control group (Fig. 7K), which was aligning with our hypothesis. Furthermore, our future studies will further investigate the repair-promoting mechanisms of POC-G/HA bone adhesive and explore its broader application scenarios.

### 3.7 In vivo evaluation of osteogenic capacity of POC-G/HA adhesive

**Fig. 8:**
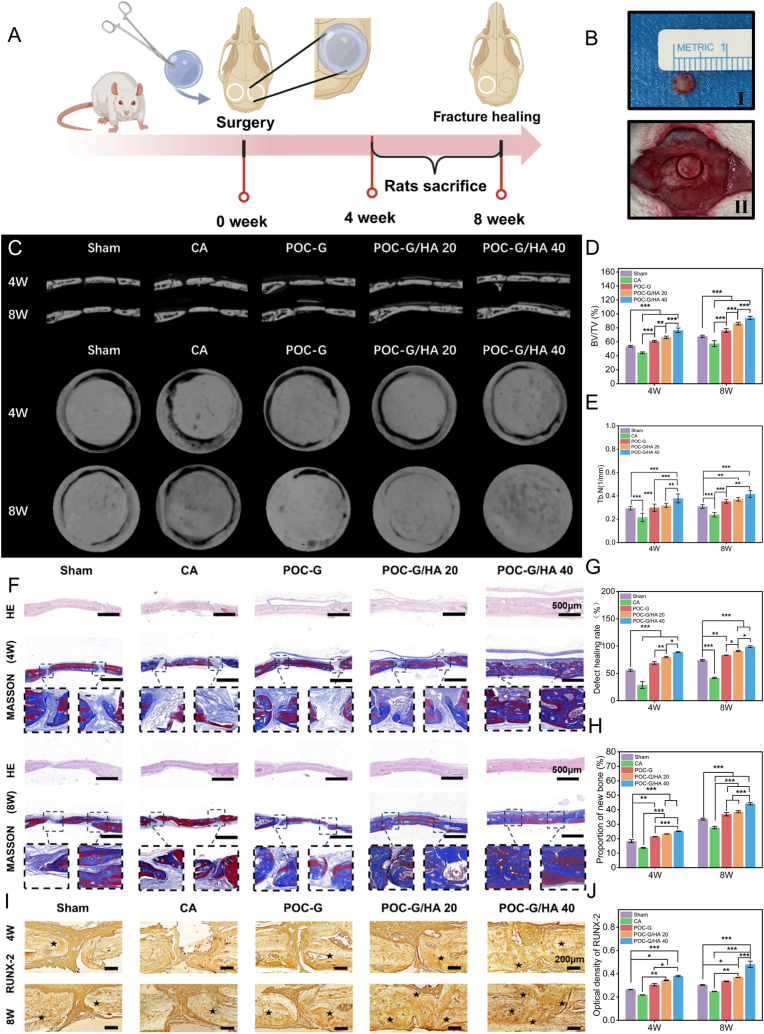
In vivo evaluation of bone regeneration in SD rats with ring-shaped cranial defects treated with POC-G/HA. (A) Schematic diagram of the cranial defect modeling, sample collection, and histological analysis procedure (B) Surgical operation demonstration diagram. (C) Representative 3D micro-CT images of the cranial defects treated with different groups at 4 and 8 weeks. Variation in the (D) BV/TV, and (E) Tb.N. parameters after 4 and 8 weeks of treatment with different groups (n = 6). (F) H&E staining and Masson's trichrome staining of the cranial defect areas at 4 and 8 weeks. The defect gap healing rate (G) and new bone formation ratio (H) after 4 and 8 weeks of treatment in different groups (n = 3). (I) Immunohistochemical staining for Runx-2 (black stars: positive areas). (J)Proportion of Runx-2-positive areas after 4 and 8 weeks of treatment in different groups (n = 3). Statistical significance: ∗p < 0.05, ∗∗p < 0.001, ∗∗∗p < 0.001 (ANOVA).

To evaluate the osteogenic capacity of the POC-G/HA bone adhesive in vivo, we created a ring-shaped cranial defect (outer diameter: 5.5 mm, inner diameter: 4 mm) in SD rats using established protocols [13,73]. The fractured cranial bones were glued together using disc-shaped POC-G/HA adhesive bandages or in situ polymerizable CA bio-glue (as the commercial control group) (Fig. 8A and B). Micro-CT to assess the bone regeneration at 4 and 8 weeks post-surgery was performed. Reconstructed 45°sagittal and vertical micro-CT views revealed relatively limited bone bridging in the Sham group (defect without treatment) (Fig. 8C). Noticeably, defects treated with CA exhibited significantly reduced bone volume at both 4 and 8 weeks as compared to Sham. We speculated this bone reduction can be related to the inherent bioinert and even cytotoxic features of CA glues, which cannot support bone re-growth and even physically impede the ingrowth of newly formed bones into the bony defects. In comparison, defects treated with POC-G/HA 40 demonstrated substantial gap closure as early as 4 weeks post-treatment. By 8 weeks, complete healing of the original ring-shaped defect was observed, indicating robust osteogenic efficacy for POC-G/HA 40. Quantitative analysis showed that POC-G/HA 40 achieved a bone volume/total volume (BV/TV) ratio of 76.52 ± 3.26 % at 4 weeks, significantly higher than Sham (53.55 ± 1.14 %), CA (44.25 ± 1.28 %), but also POC-G (60.78 ± 1.23 %) and POC-G/HA 20 (66.49 ± 11.74 %). Remarkably, at 8 weeks, the BV/TV ratio for POC-G/HA 40 even reached up to 94.20 ± 2.23 %, demonstrating statistically superior osteogenic inductivity as compared to other groups (Fig. 8D). Furthermore, trabecular number (Tb.N), another crucial parameter for assessing bone quality, reached 0.38 ± 0.04/mm in the POC-G/HA 40 group at 4 weeks, showing significant superiority over both Sham (0.29 ± 0.01/mm) and CA (0.21 ± 0.04/mm). By 8 weeks, the Sham group achieved 0.31 ± 0.02/mm while CA only reached 0.24 ± 0.02/mm. In contrast, the POC-G, POC-G/HA 20, and POC-G/HA 40 groups demonstrated markedly better performance with values of 0.35 ± 0.02/mm, 0.37 ± 0.02/mm, and 0.41 ± 0.03/mm respectively (Fig. 8E).

Further H&E and Masson's trichrome staining methods were employed to histologically assess new bone quality. At 4 and 8 weeks post-surgery, the Sham group displayed limited bone regeneration within the defect, while the CA group exhibited even less new bone regrowth (Fig. 8F). Quantitative defect closure analysis revealed significantly lower healing rates at 4 weeks in Sham (55.66 ± 2.04 %) and CA (28.34 ± 6.34 %) compared to POC-G/HA 40 (88.96 ± 0.73 %), with POC-G/HA 20 and POC-G showing intermediate values. By 8 weeks, POC-G/HA 40 achieved a healing rate of 98.85 ± 1.62 %, surpassing that of Sham (74.12 ± 1.36 %), CA (41.66 ± 1.26 %), POC-G (83.36 ± 0.32 %), and POC-G/HA 20 (91.22 ± 1.10 %) (Fig. 8G). Masson's trichrome staining distinguishes osteoid (blue) from mature bone matrix (red), which showed limited new bone in Sham at 4 weeks, with the defect largely unhealed at 8 weeks (Fig. 8F). CA-treated defects displayed minimal new bone and abundant fibrous tissue at both time points. Conversely, POC-G/HA 40 demonstrated enormous woven bone formation at 4 weeks and near-complete healing by 8 weeks. Quantitatively, new bone area in POC-G/HA 40 was 25.15 ± 0.21 % at 4 weeks, increasing to 44.23 ± 0.90 % at 8 weeks—significantly higher than in Sham (33.53 ± 0.66 %), CA (27.71 ± 0.85 %), POC-G (36.96 ± 1.21 %), and POC-G/HA 20 (38.59 ± 0.72 %) (Fig. 8H).

Further immunohistochemical staining of osteogenic marker Runx-2 revealed that at 4 and 8 weeks post-surgery, the Sham group exhibited limited, scattered Runx-2-positive areas, while the CA group showed minimal staining positivity (Fig. 8I), indicating impaired osteogenic differentiation. In contrast, POC-G/HA 40 displayed extensive Runx-2-positive signals within defect-site. Quantitatively, POC-G/HA 40 achieved 38.11 ± 0.64 % positively stained tissue areas at 4 weeks—significantly exceeding Sham (26.42 ± 0.14 %). By 8 weeks, this increased to 48.13 ± 2.74 %, outperforming Sham (30.36 ± 0.31 %), CA (24.75 ± 0.17 %), POC-G (33.50 ± 0.30 %), and POC-G/HA 20 (37.02 ± 0.11 %) (Fig. 8J). These results confirmed the POC-G/HA adhesive's capacity to induce osteogenesis at bone defective sites.

**Fig. 9:**
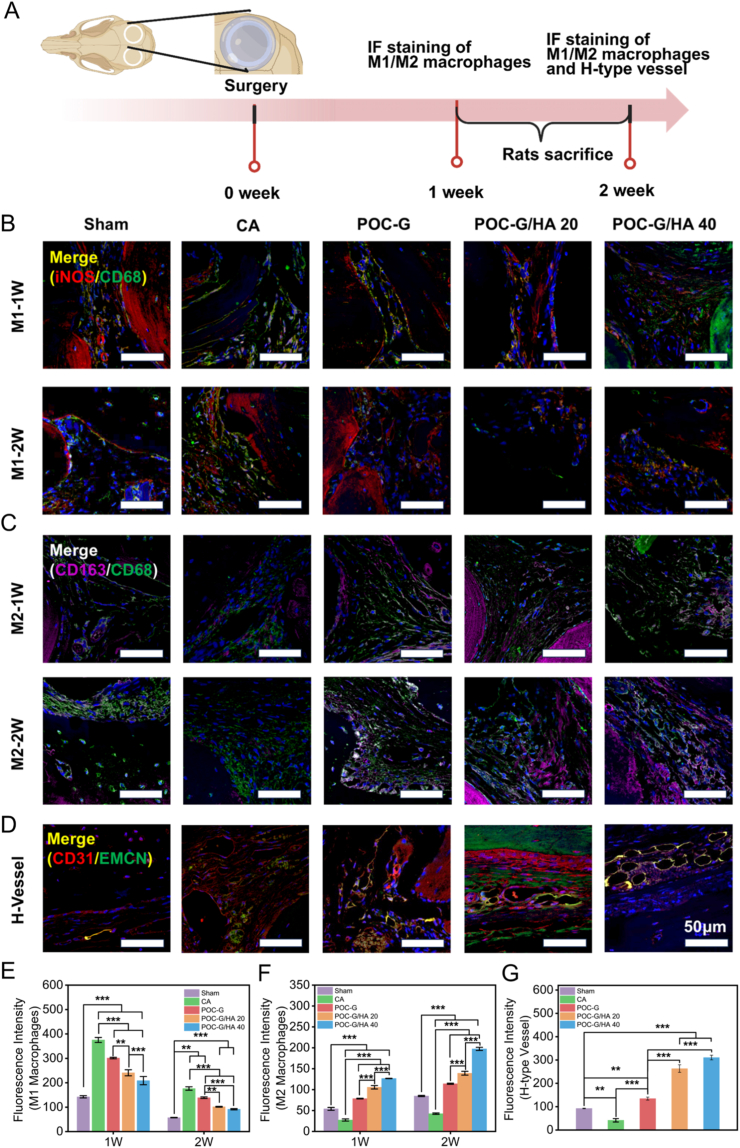
Immunofluorescence staining of SD rat cranial defect treated by various POC-G/HA and CA. (A) Schematic diagram of the immunofluorescence experimental procedure. (B) Immunofluorescence staining of M1 macrophage polarization (green: CD68^+^, red: iNOS+, yellow: merge). (C) Immunofluorescence staining of M2 macrophage polarization (green: CD68^+^, pink: CD163+, white: merge). (D) Immunofluorescence staining of H-type blood vessels (red: CD31^+^, green: EMCN+, yellow: merge). Quantitative analysis of fluorescence intensity for M1 macrophages (E), M2 macrophages (F) and H-type vessels (G) (n = 3). Statistical significance: ∗p < 0.05, ∗∗p < 0.001, ∗∗∗p < 0.001 (ANOVA).

Given the in vitro demonstrations of angiogenic and immunomodulatory capacities of POC-G/HA composites, we further evaluated their effects on local immune responses and vascularization during bone repair in the animal experiments. To this end, we first evaluated the presence of local macrophages and their polarization upon the treatment of POC-G/HA composites via immunofluorescent co-staining for M1 (iNOS^+^/CD68^+^) and M2 (CD163^+^/CD68^+^) phenotypes of macrophages. CA induced substantially higher proportion of M1 versus M2 macrophages locally at the defect sites after 1 week post-surgery (Fig. 9B and C), indicating the severe pro-inflammatory microenvironment generated by the presence of CA locally. Specifically, at week 1 there were significantly higher fluorescence intensity for M1 macrophages in the CA group (375.36 ± 10.30 a.u.) that of Sham group (142.40 ± 4.62 a.u.) (Fig. 9E). Meanwhile, fluorescence intensity for M2 macrophages in the CA group (27.37 ± 2.67 a.u.) was significantly lower than Sham (54.01 ± 3.44 a.u.). In comparison, POC-G/HA 40 induced M1 signals of 208.79 ± 16.78, which was between CA and Sham (p < 0.001). Regarding M2 regulation, POC-G/HA composites demonstrated a concentration-dependent anti-inflammatory effect, with M2 fluorescence intensity for POC-G (78.59 ± 0.87 a.u.), POC-G/HA 20 (105.69 ± 4.11 a.u.) and POC-G/HA 40 (126.70 ± 0.43 a.u.) (p < 0.001, Fig. 9F). Similar trends were also observed for samples at week 2 post-implantation. These results confirmed POC-G/HA composites were capable to establish a pro-regenerative local microenvironment for bone repair through superior immunomodulation.

Angiogenic capacity was first assessed through histological identification of H-type vessels—specialized microvasculature characterized by CD31^+^/EMCN^+^ co-expression that coupled angiogenesis with osteogenesis [74,75]. Immunofluorescence co-staining of CD31/EMCN in cranial specimens at 2 weeks post-implantation revealed sparse co-localized (yellow) tubular structures in Sham, while CA exhibited negligible signal. In contrast, POC-G/HA 40 displayed the most pronounced network of co-localized tubules, outperforming both POC-G/HA 20 and POC-G (Fig. 9D). Further quantitative analysis revealed that POC-G/HA 40 possessed remarkable angiogenic potential, demonstrating a fluorescence intensity of 310.61 ± 9.89 a.u. (Fig. 9G). This value was significantly higher than those observed in the Sham (92.42 ± 1.46 a.u.) and CA (41.69 ± 7.24 a.u.) groups (p < 0.001), followed by the POC-G (134.60 ± 6.44 a.u.) and POC-G/HA 20 (263.22 ± 16.25 a.u.). The formation of H-type vessels may facilitate local recruitment of osteoprogenitor cells and improve nutrient exchange, thereby supporting osteogenesis. Collectively confirming the POC-G/HA adhesive's capacity to potentiate bone regeneration through enhanced vascularization. The newly formed vessels facilitated tissue regeneration through dual functions: 1) recruiting fibroblasts that formed supportive matrices for vascular ingrowth; 2) attracting mesenchymal stem cells that differentiated into osteoblasts; and 3) mobilizing macrophages for debris clearance [64]. This coordinated cellular response collectively promoted osteogenic microenvironments.

In general, the in vivo study demonstrated the feasibility of using POC-G/HA adhesive bandages towards the structural integration and osteogenesis of fractured bones, and confirmed the bioactivity but also the osteogenesis after long-term in vivo investigation. Specifically, the strong adhesiveness of the composite adhesives can instantaneously bridge the bone fragments together and avoid introducing physical barriers between defective bones; this is essential for further bone regrowth and bridging. Biochemically, the presence of osteogenic HA minerals and the degraded citrates from the composites can collectively promote local mesenchymal stem cells towards osteogenic differentiation (Fig. 8I). Although the citrates from POC can slightly lower local pH, they have been demonstrated biocompatible and capable to induce bone regeneration [21,23,57]. Moreover, HA can further support bone formation, repair, and regeneration [4,47]. More importantly, our results indicated the considerable immunoregulatory efficacy of POC-G/HA composites by suppressing M1 macrophage activation and reducing the secretion of pro-inflammatory cytokines (IL-1β and TNF-α), but also promoting macrophage polarization toward the pro-regenerative M2 phenotype. This can establish a favorable immune microenvironment to support bone regeneration [[62], [63], [64], [65]]. Furthermore, previous studies proved that citrates can contribute to vascular formation, which aided to recruit necessary cells, nutrients, and mass exchange required for bone regeneration [21,76]. Our findings affirmed this, showing that POC-G/HA enhanced angiogenesis through upregulating CD31 and EMCN gene expression and VEGF secretion in endothelial cells.

In clinical practice, when fractures occur in weight-bearing bones, metal internal fixation devices remain essential for maintaining structural stability due to the significant load-bearing demands [[77], [78], [79]]. Therefore, bone adhesives primarily serve as supplementary fixation tools, capable of reducing displacement at the fracture site and enhancing the contact interface between fracture surfaces, thereby promoting bone healing. Moreover, the integration of the adhesive with surrounding bone tissue further stabilizes the fracture interface, reducing the need for prolonged robust adhesion by the material itself after the initial stage as healing progresses [80]. Based on this functional role, our research focuses on evaluating the material's adhesive properties and osteogenic capabilities. Following the methods described by Duan et al. and Xu et al. [13,73], we selected the rat cranial ring-shaped defect model. Compared to traditional models (such as femoral fractures or long bone segmental defects), the cranial ring-shaped defect model eliminates the need for additional metal internal fixation devices, thereby reducing surgical complexity. This enables a clear assessment of whether the adhesive can maintain the stability of bone fragments and the extent of its contribution to bone generation. Simultaneously, we acknowledge the limitations of this model. It cannot simulate the mechanical loading environment of long bones and therefore cannot demonstrate the adhesive's performance under physiological loading conditions. Consequently, in future research, we plan to incorporate femoral fracture or long bone segmental defect models to further evaluate the performance of the adhesive under mechanical loading conditions.

Cyanoacrylate (CA) bio-glue is a widely used commercial tissue adhesive capable of rapidly bonding to bone tissues, including cranial and articular bones. Previous studies have demonstrated that CA bio-glue forms a dense layer on bone surfaces, providing mechanical stability; however, its flowability prior to complete solidification can create a barrier that impedes the inward migration of bone cells [13,81]. Moreover, its chemical inertness and cytotoxic degradation byproducts, including cyanoacetic acid and formaldehyde, can provoke local inflammation and inhibit regeneration [[82], [83], [84]]. By comparison, the POC-G/HA bone adhesives exhibit several distinct advantages. First, its strong adhesion, mechanical stability, and moderate degradation rate ensure interfacial stability and structural integrity of fractured bones, thereby preventing loss of mechanical support and promoting bone regrowth. Second, it possesses intrinsic osteogenic capacity without the need for exogenous osteogenic therapeutics such as growth factors or biomolecules, simplifying both preparation and application. Third, its bandage-like formulation mimics the periosteum, overcoming the limitations of flowable bio-glues that could infiltrate in defect gaps and spatially hinder bone bridging. In addition, POC-G/HA bone adhesives offer ease of application, environmentally benign synthesis, cost-effectiveness, and robust bone regenerative performance, highlighting their translational potential in bone repair. Collectively, our findings demonstrate that POC-G/HA adhesives combine essential functions for bone fixation and reconstruction with bioactive properties, including osteogenesis, immunomodulation, and angiogenesis. These multifunctional characteristics position them as a promising new generation of bone repair biomaterials with advanced therapeutic potential.

## 4 Conclusion

In this study, we developed a novel class of pressure-sensitive POC-G/HA bone adhesive bandages capable of providing rapid and stable bone adhesion under physiological moist conditions. The experimental results show that the prepared bone adhesive bandage has enhanced its mechanical properties and stable bone adhesion in a wet environment via the organic-inorganic composite. Beyond its adhesive function, the composite significantly promoted bone regeneration by inducing stem cell osteogenic differentiation, modulating macrophage polarization toward the anti-inflammatory M2 phenotype, and stimulating angiogenesis, as validated in a rat ring-shaped cranial defect model. Both in vitro and in vivo evaluations demonstrated the superior biocompatibility and controllable biodegradability of POC-G/HA. Our POC-G/HA composite bone adhesives outperform currently available bone bio-glues by offering stronger interfacial adhesion, intrinsic osteoinductivity, and favorable immunomodulatory and angiogenic properties, meanwhile providing practical advantages such as ease of application, environmentally benign synthesis, and cost-effectiveness. Collectively, these multifunctional performances position POC-G/HA as a promising next-generation bone repair material with substantial translational potential for clinical applications in fracture fixation and bone reconstruction.

## CRediT authorship contribution statement

**Puzhou Lei:** Writing – original draft, Software, Project administration, Methodology, Formal analysis, Data curation, Conceptualization. **Shuya Wang:** Writing – original draft, Software, Project administration, Methodology, Formal analysis, Data curation, Conceptualization. **Kaiwen Chen:** Writing – original draft, Software, Project administration, Methodology, Funding acquisition, Formal analysis. **Linghanqing Wang:** Validation, Investigation. **Yi Shen:** Software, Methodology. **Huize Zhong:** Methodology, Formal analysis. **Sida Chen:** Validation, Formal analysis. **Xin Su:** Methodology, Formal analysis. **Yu Zhao:** Validation, Investigation. **Huanan Wang:** Writing – review & editing, Supervision, Resources, Methodology, Funding acquisition, Formal analysis, Conceptualization. **Lei Li:** Writing – review & editing, Supervision, Resources, Methodology, Funding acquisition, Formal analysis, Conceptualization.

## Ethics approval and consent to participate

All procedures followed the National Institute of Health Guide for the Care and Use of Laboratory Animals and were approved by The Medical Ethics Committee of Shengjing Hospital of China Medical University (approved number: 2023PS1248K).

## Declaration of competing interest

The authors declare that they have no known competing financial interests or personal relationships that could have appeared to influence the work reported in this paper.

## Data Availability

Data will be made available on request.
