# Supplementary material for: Pressure-sensitive composite bandages with high toughness, self-healability and strong tissues adhesion for rapid bone fixation and accelerated osteogenesis via immunomodulation and neovascularization
Source: Mater Today Bio. 2025 Dec 23;36:102730. doi: 10.1016/j.mtbio.2025.102730 (PMC12813315; doi:10.1016/j.mtbio.2025.102730)
Supplement: Multimedia component 1 [file mmc1.docx]

# Supporting Information

**Table S1.** Comprehensive properties of recent adhesives stability and asymmetric-adhesion.

|  | **Adhesive strength (kPa)** | | **Repeatable adhesion** | **Operation time (s)** | | **Elongation at break (%)** | **Self-healing** | **Swelling ratio (%)** | **X = Convenient adhesion** | **Y = Mechanical stability** |
| --- | --- | --- | --- | --- | --- | --- | --- | --- | --- | --- |
| **HB-PTN**[1] | | ＞ 200 | - | | ＜ 100 | ＜ 100 | √ | ＜ 20 | 2 | 1 |
| **GelDex-AMBGN**[2] | | ＜ 200 | √ | | ＞ 1000 | ＞ 100 | √ | ＞ 300 | 1 | 2 |
| **SF@TA@HA**[3] | | ＞ 900 | - | | ＞ 100 | ＞ 100 | √ | ＞ 60 | 1 | 2 |
| **POx-OH-Ale**[4] | | ＜ 200 | - | | ＞ 100 | - | √ | ＞ 1000 | 0 | 1 |
| **RPO-H**[5] | | 20-30 | - | | ＞ 100 | ＜ 100 | √ | ＜ 20 | 0 | 2 |
| **GeIMA/Arg/nHAMA**[6] | | ＞ 500 | - | | - | ＞ 100 | - | ＞ 1000 | 1 | 1 |
| **GeIMA/SilMA/GelDA/GO**[7] | | ＜ 20 | - | | 300 | ＜ 100 | - | ＞ 1000 | 0 | 0 |
| **iCMBA/HA**[8] | | ＜ 200 | - | | ＞ 100 | ＜ 100 | - | ＞ 100 | 0 | 0 |
| **POC-G/HA (This work)** | | ＞ 500 | √ | | ＜ 60 | ＞ 400 | √ | ＜ 4 | 3 | 3 |

If the adhesive strength is more than 200 kPa, the score of adhesives was 1, otherwise it is 0;

If the adhesive is repeatable, the score of adhesives was 1, otherwise it is 0;

If the operation time of adhesives is less than 100s, the score of adhesives was 1, otherwise it is 0;

If the elongation at break of adhesives is more than 100%, the score of adhesives was 1, otherwise it is 0;

If the adhesive has self-healing ability, the score of adhesives was 1, otherwise it is 0;

If the swelling ratio is less than 20 %, the score of adhesives was 1, otherwise it is 0.

X: Convenient adhesion = the sum score of "adhesive strength", "repeatable adhesion" and "operation time".

Y: Mechanical stability = the sum score of "elongation at break", "swelling ratio" and "self-healing".

**References:**

[1] C. Zhou, C. Liu, D. Sha, L. Sun, C. Liu, Y. Yuan, Homogeneous “Hard-Soft” Biphasic Bone Adhesives Promote Comminuted Fracture Healing through Interfacial Adaptation and Mechanical Property Maintenance, Advanced Materials 37 (2025) 2502598. https://doi.org/10.1002/adma.202502598.

[2] J. Tang, K. Xi, H. Chen, L. Wang, D. Li, Y. Xu, T. Xin, L. Wu, Y. Zhou, J. Bian, Z. Cai, H. Yang, L. Deng, Y. Gu, W. Cui, L. Chen, Flexible Osteogenic Glue as an All-In-One Solution to Assist Fracture Fixation and Healing, Advanced Functional Materials 31 (2021) 2102465. https://doi.org/10.1002/adfm.202102465.

[3] S. Bai, X. Zhang, X. Lv, M. Zhang, X. Huang, Y. Shi, C. Lu, J. Song, H. Yang, Bioinspired Mineral–Organic Bone Adhesives for Stable Fracture Fixation and Accelerated Bone Regeneration, Advanced Functional Materials 30 (2020) 1908381. https://doi.org/10.1002/adfm.201908381.

[4] M.J. Sánchez-Fernández, J. Rutjes, R.P. Félix Lanao, J.C.M.E. Bender, J.C.M. van Hest, S.C.G. Leeuwenburgh, Bone-Adhesive Hydrogels Based on Dual Crosslinked Poly(2-oxazoline)s, Macromol Biosci 21 (2021) e2100257. https://doi.org/10.1002/mabi.202100257.

[5] X. Xing, Z. Gong, C. Chen, Y. Lin, P. Liu, T. Xiao, H. Yu, Y. Li, Y. Lin, G. Tan, C. Ning, Z. Wu, L. Wang, L. Zhou, Injectable bioresponsive bone adhesive hydrogels inhibit NLRP3 inflammasome on demand to accelerate diabetic fracture healing, Biomaterials 317 (2025) 123059. https://doi.org/10.1016/j.biomaterials.2024.123059.

[6] Y. Yang, T. Xu, Q. Zhang, Y. Piao, H.P. Bei, X. Zhao, Biomimetic, Stiff, and Adhesive Periosteum with Osteogenic–Angiogenic Coupling Effect for Bone Regeneration, Small 17 (2021) 2006598. https://doi.org/10.1002/smll.202006598.

[7] X. Sun, J. Yang, J. Ma, T. Wang, X. Zhao, D. Zhu, W. Jin, K. Zhang, X. Sun, Y. Shen, N. Xie, F. Yang, X. Shang, S. Li, X. Zhou, C. He, D. Zhang, J. Wang, Three-dimensional bioprinted BMSCs-laden highly adhesive artificial periosteum containing gelatin-dopamine and graphene oxide nanosheets promoting bone defect repair, Biofabrication 15 (2023) 025010. https://doi.org/10.1088/1758-5090/acb73e.

[8] D. Xie, J. Guo, M.R. Mehdizadeh, R.T. Tran, R. Chen, D. Sun, G. Qian, D. Jin, X. Bai, J. Yang, Development of injectable citrate-based bioadhesive bone implants, J. Mater. Chem. B 3 (2014) 387–398. https://doi.org/10.1039/C4TB01498G.

**Table S2**. RT-PCR Primer Information

| BMP2 | Forward Primer（F） | AACGAGAAAAGCGTCAAGCC |
| --- | --- | --- |
|  | Reverse Primer（R） | AGGTGCCACGATCCAGTCAT |
| Alp | Forward Primer（F） | AGGGTGGGTTTCTCTCTTGG |
|  | Reverse Primer（R） | CATGATGGTTGCAGGGTCTG |
| Runx2 | Forward Primer（F） | TCTGACACGGCTTTACCTGT |
|  | Reverse Primer（R） | AGCAAAGAGGCCGCATTAAG |
| GAPDH | Forward Primer（F） | AAGATGGTGAAGGTCGGTGT |
|  | Reverse Primer（R） | GCTTCCCATTCTCAGCCTTG |


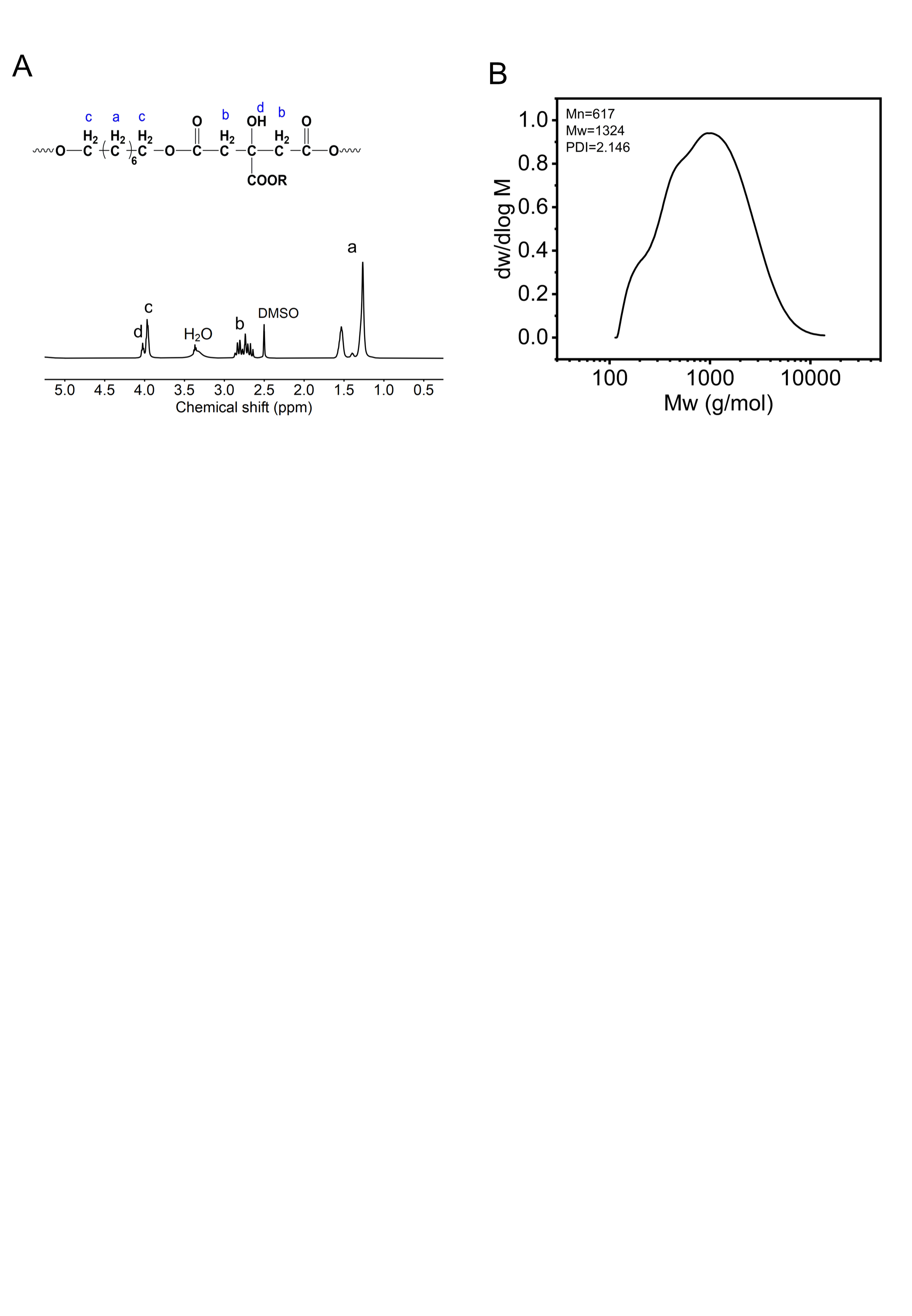


**Figure S1.** (A) Synthesis equation and 1H NMR spectrum of pre-POC. (B) Molecular weight characterization of pre-POC.


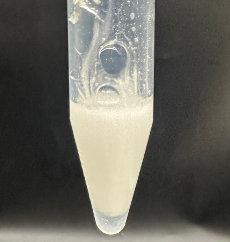

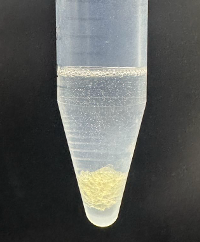

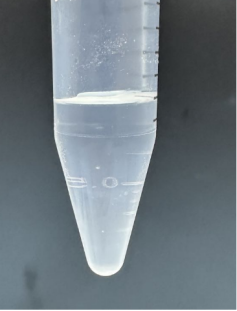


**Figure S2.** POC/gelatin dissolves in water (left), ethanol (middle), and ethanol-water solution (right).


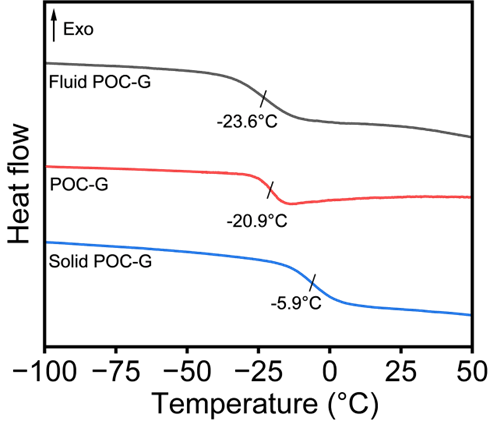


**Figure S3.** Differential scanning calorimetry (DSC) of amphiphilic copolymers under different reaction times. Short lines are used to mark the glass transition temperatures of each sample.


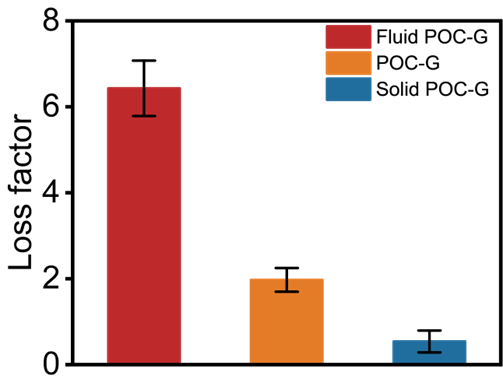


**Figure S4.** Quantification of loss factor under different response times (fluid POC-G, 12h; POC-G, 24 h; solid POC-G, 48 h).


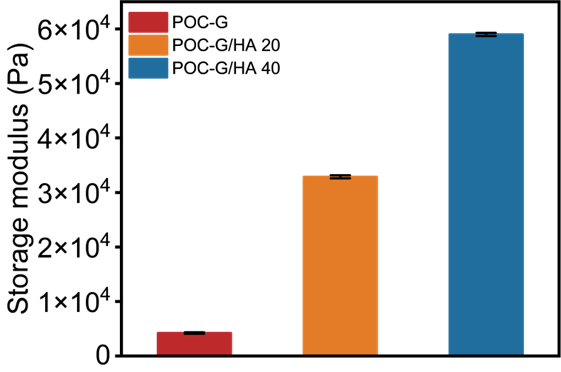


**Figure S5.** Quantification of loss factor of POC-G, POC-G/HA 20 and POC-G/HA 40.


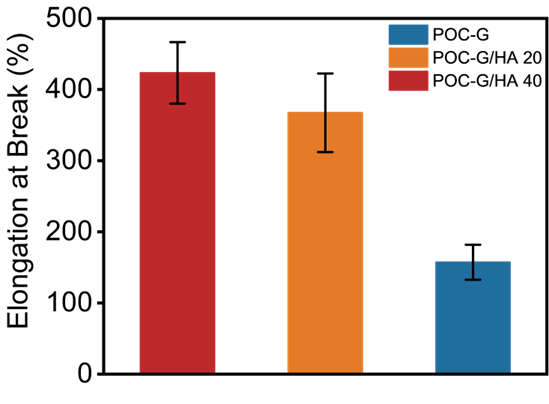


**Figure S6.** Elongation at break of POC-G, POC-G/HA 20 and POC-G/HA 40.


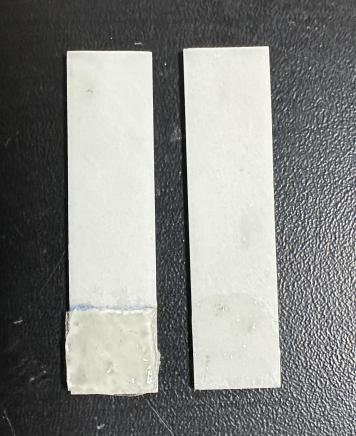

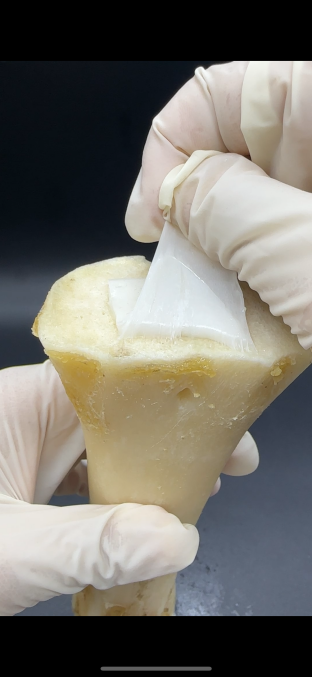


**Figure S7.** Photographs of the lap shear failure mode (adhesive failure) of POC-G/HA 20 bone adhesive (left) and its detachment from the bone tissue surface (right).


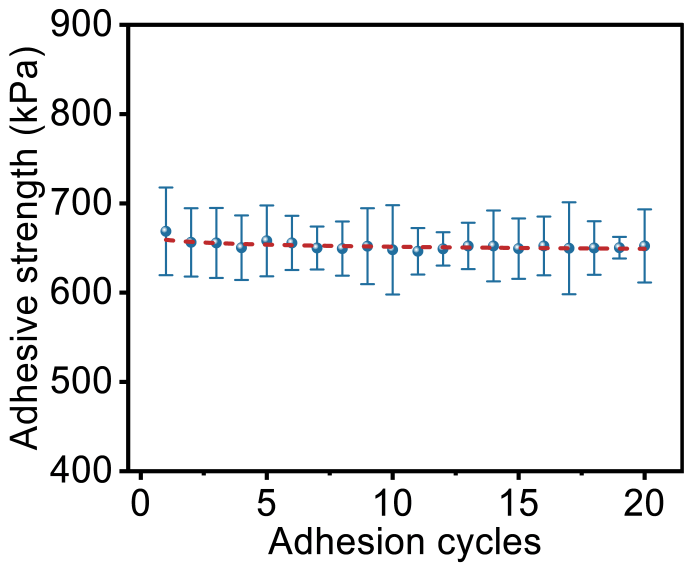


**Figure S8.** The cyclic tensile peel test, the adhesive strength of POC-G/HA 20 after repeated adhesion with bone slices 20 times (n=3).


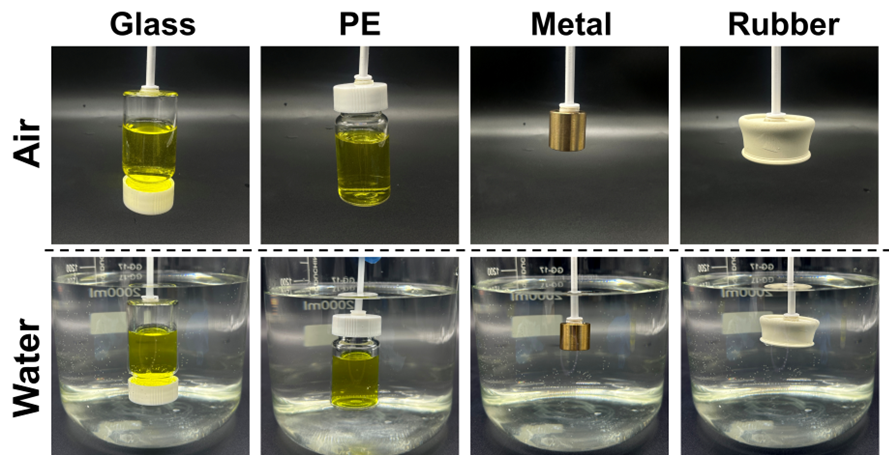


**Figure S9.** Digital photos of POC-G/HA 20 patch adhering to various substrates (glass, PE, metal and rubber) in water and air.


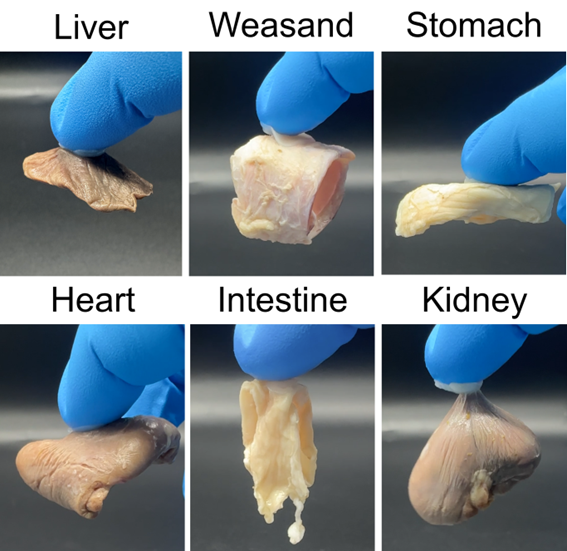


**Figure S10.** Photographs of the adhesion of the POC-G/HA 20 adhesive to a variety of tissues, including liver, weasand, stomach, heart, small intestine, and kidney.


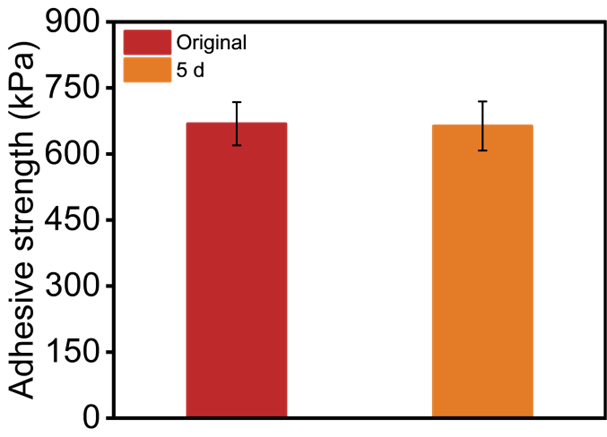


**Figure S11.** The adhesive strength of the POC-G/HA 20 adhesive bandage after being immersed in SBF for 5 days.


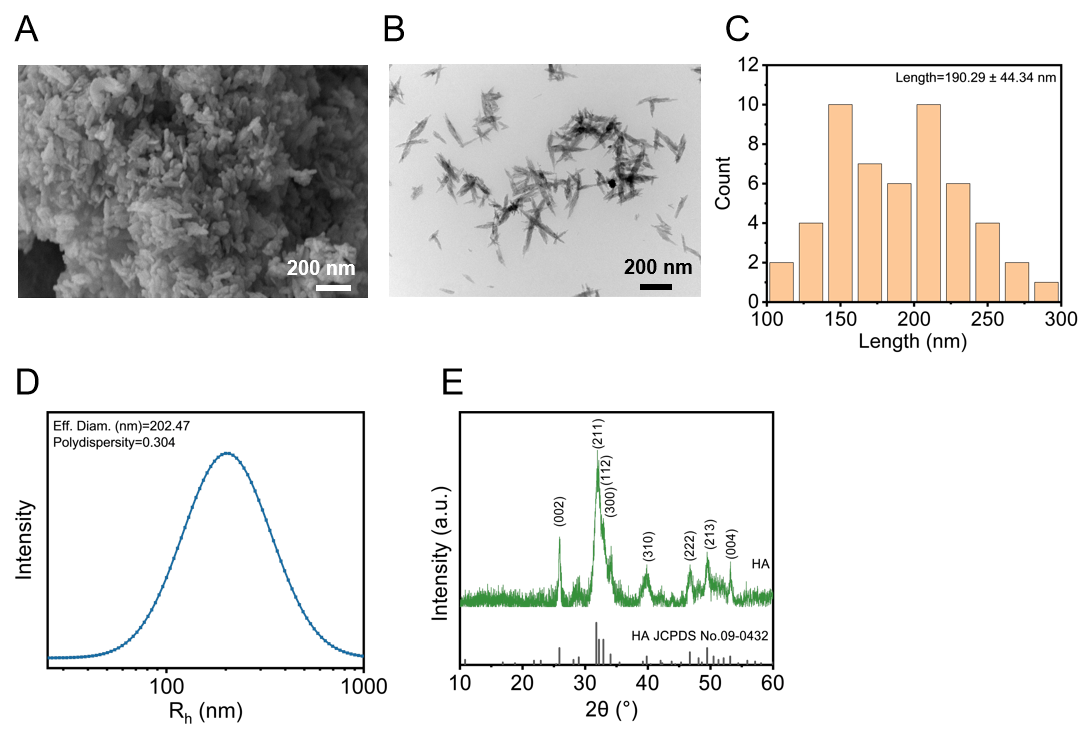


**Figure S12.** Characterization of the hydroxyapatite. (A) SEM image of HA nanoparticles. (B) TEM image of HA nanoparticles. (C) The number-average size distribution of lengths of HA nanoparticles. (D) Particle size of HA nanoparticles dispersed in ethanol. (E) Diffraction patterns of HA nanoparticles and HA standard diffraction pattern of XRD test.


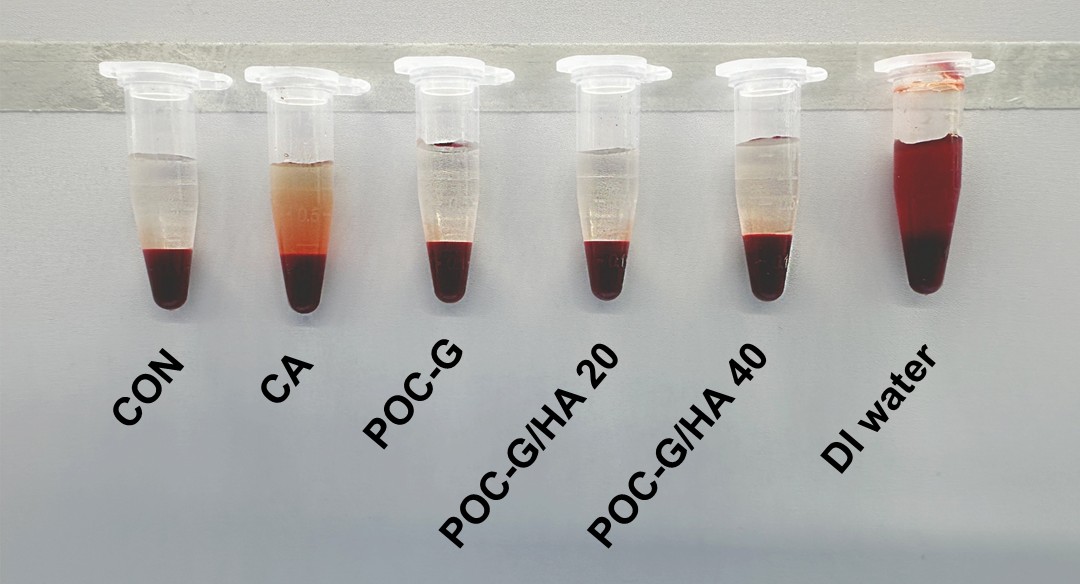


**Figure S13.** Hemolysis test result of POC-G/HA bone adhesive and CA bioglue.


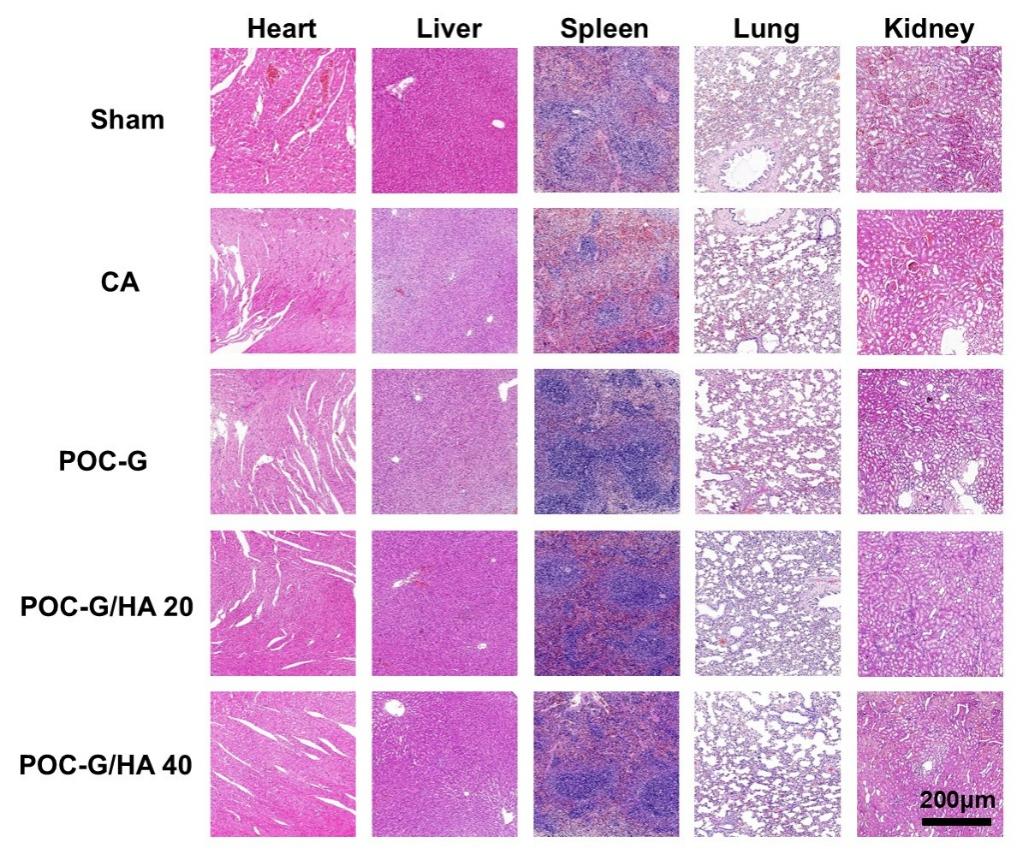


**Figure S14.** HE staining of heart, liver, spleen, lung and kidney tissues in SD rats at 8 weeks post-implantation of POC-G/HA bone adhesive and CA bioglue.


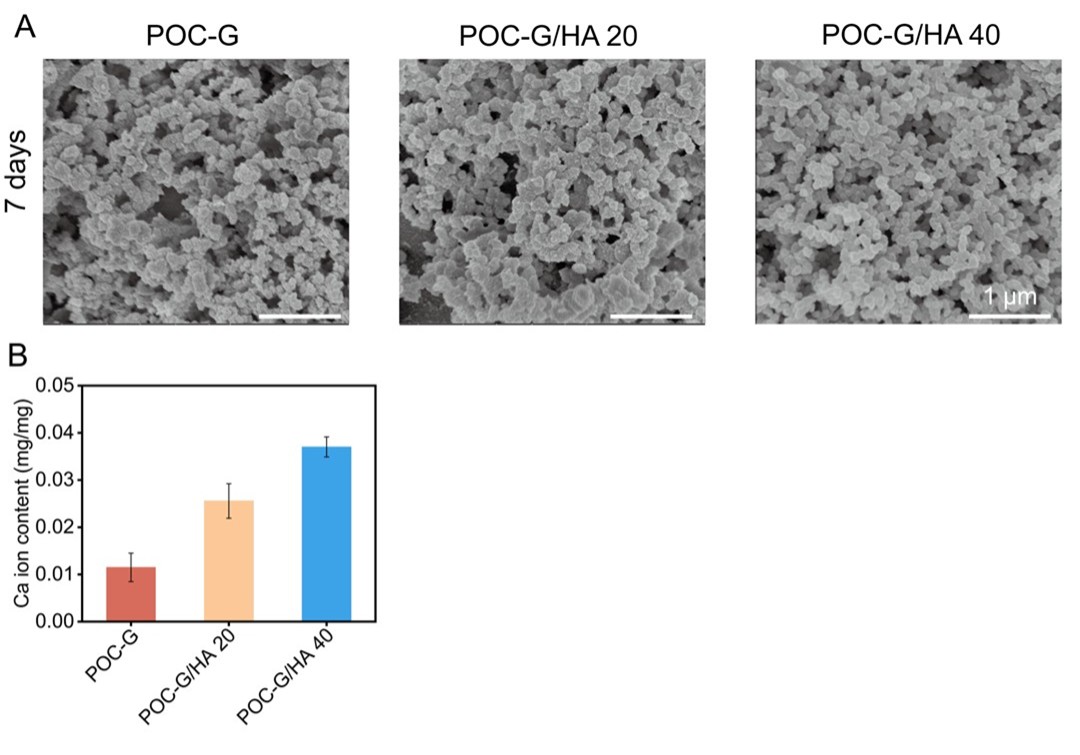


**Figure S15.** In vitro mineralization and degradation of POC-G/HA adhesive patches. (A) Scanning electron microscopy images of adhesive patches before and after immersion in 3x-SBF. (B) Quantification of the increase in calcium ion content of the adhesive patches after mineralization.


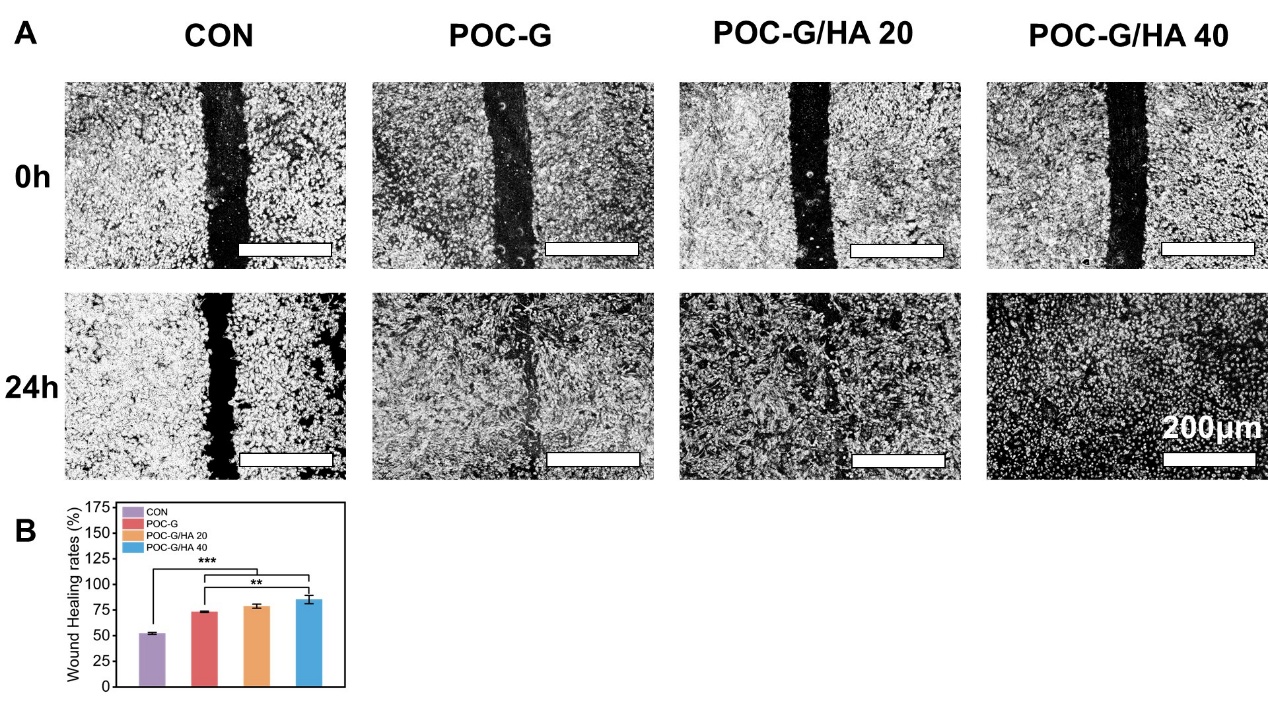


**Figure S16.** (A) Images of migration by HUVECs after co-cultured with POC-G/HA for 24 hours. (B) Quantification of wound healing rates of HUVECs.
